# TRPM7 is the central gatekeeper of intestinal mineral absorption essential for postnatal survival

**DOI:** 10.1073/pnas.1810633116

**Published:** 2019-02-15

**Authors:** Lorenz Mittermeier, Lusine Demirkhanyan, Benjamin Stadlbauer, Andreas Breit, Camilla Recordati, Anne Hilgendorff, Masayuki Matsushita, Attila Braun, David G. Simmons, Eleonora Zakharian, Thomas Gudermann, Vladimir Chubanov

**Affiliations:** ^a^Walther-Straub Institute of Pharmacology and Toxicology, Ludwig-Maximilians-Universität München, Munich 80336, Germany;; ^b^Department of Cancer Biology and Pharmacology, University of Illinois College of Medicine, Peoria, IL 61605;; ^c^Mouse and Animal Pathology Laboratory, Filarete Foundation, 20139 Milan, Italy;; ^d^Center for Comprehensive Developmental Care, Integrated Social Pediatric Center, Dr. von Hauner Children’s Hospital, University Hospital, Ludwig-Maximilians-Universität München, Munich 80337, Germany;; ^e^Comprehensive Pneumology Center, Munich 80337, Germany;; ^f^German Center for Lung Research, Munich 80337, Germany;; ^g^Department of Molecular and Cellular Physiology, Graduate School of Medicine, University of the Ryukyus, Okinawa 903-0215, Japan;; ^h^Institute of Experimental Biomedicine, University Hospital Würzburg, Würzburg 97080, Germany;; ^i^School of Biomedical Sciences, University of Queensland, QLD 4072, Australia;; ^j^Munich Heart Alliance, Munich 80336, Germany

**Keywords:** TRP channels, TRPM7, zinc, calcium, magnesium

## Abstract

Zn^2+^, Mg^2+^, and Ca^2+^ are essential minerals required for a plethora of metabolic processes and signaling pathways. Different categories of cation-selective channels and transporters are therefore required to tightly control the cellular levels of individual metals in a cell-specific manner. However, the mechanisms responsible for the organismal balance of these essential minerals are poorly understood. Herein, we identify a central and indispensable role of the channel-kinase TRPM7 for organismal mineral homeostasis. The function of TRPM7 was assessed by single-channel analysis of TRPM7, phenotyping of TRPM7-deficient cells in conjunction with metabolic profiling of mice carrying kidney- and intestine-restricted null mutations in *Trpm7* and animals with a global “kinase-dead” point mutation in the gene. The TRPM7 channel reconstituted in lipid bilayers displayed a similar permeability to Zn^2+^ and Mg^2+^. Consistently, we found that endogenous TRPM7 regulates the total content of Zn^2+^ and Mg^2+^ in cultured cells. Unexpectedly, genetic inactivation of intestinal rather than kidney TRPM7 caused profound deficiencies specifically of Zn^2+^, Mg^2+^, and Ca^2+^ at the organismal level, a scenario incompatible with early postnatal growth and survival. In contrast, global ablation of TRPM7 kinase activity did not affect mineral homeostasis, reinforcing the importance of the channel activity of TRPM7. Finally, dietary Zn^2+^ and Mg^2+^ fortifications significantly extended the survival of offspring lacking intestinal TRPM7. Hence, the organismal balance of divalent cations critically relies on one common gatekeeper, the intestinal TRPM7 channel.

## Significance

Zn^2+^, Mg^2+^, and Ca^2+^ are the most abundant divalent metals in mammals. Different categories of cation-selective channels and transporters are thought to control the levels of individual metals in a cell-specific manner. However, the mechanisms responsible for the organismal balance of these minerals are poorly understood. Using genetic mouse models together with biophysical and biochemical analysis, we show that the channel-kinase TRPM7 is a master regulator of the organismal balance of divalent cations. TRPM7 activity is primarily required in the intestine, while TRPM7 function in the kidney—commonly thought to be essential—is expendable. Hence, against current thinking, organismal balance of multiple divalent cations predominantly relies on a common gatekeeper, TRPM7, rather than on individual specialized channels/transporters.

Zn^2+^, Mg^2+^, and Ca^2+^ are vital divalent cations implicated in a myriad of physiological and pathophysiological processes (1–3). In the body, less than 1% of the total content of these metals is present in the circulation, and the overall balance is tightly controlled by intestinal absorption, renal excretion, and backup storage in bones. The ability of the body to tightly regulate the circulating levels of Zn^2+^, Mg^2+^, and Ca^2+^ is essential for normal development and overall health as illustrated by heritable human diseases such as transient neonatal zinc deficiency (TNZD) (4), hypomagnesemia with secondary hypocalcemia (HSH) (5), and vitamin D-dependent rickets (6).

Early studies posited that the nutritional uptake of Zn^2+^, Mg^2+^, and Ca^2+^ is maintained by intestinal enterocytes and consists of an apical entry and a basolateral extrusion step (1–3). Historically, specialized channels are thought to underlie these regulatory steps in a cation-specific fashion. Thus, the Ca^2+^-selective TRPV6 channel was proposed to function as a major player in intestinal Ca^2+^ uptake (7). However, *Trpv6* null mice did not display any significant reduction of serum Ca^2+^ levels and showed only modestly diminished (7) or even unchanged intestinal Ca^2+^ absorption (8, 9), indicating that additional absorption pathways must exist.

Twenty-four members of the solute carriers of family 30 (Slc30a1-10 or ZnT1-10) and family 39 (Slc39a1-14 or Zip1-14) are assumed to control cytosolic Zn^2+^ levels in a cell-specific manner (2). However, to the best of our knowledge, only Zip4 has been associated with a genetic defect of intestinal Zn^2+^ uptake triggering organismal Zn^2+^ deficiency. Thus, mutations in the human *ZIP4* gene cause acrodermatitis enteropathica (AE) (4). Infants with AE fed breast milk containing particularly high amounts of Zn^2+^ are asymptomatic. Soon after weaning, however, patients with AE display skin lesions and other symptoms caused by Zn^2+^ deficiency (4). Conditional intestine-restricted inactivation of *Zip4* in mice recapitulated these AE symptoms (10). The normal physical appearance of breast-fed patients with AE and *Zip4*-deficient pups was interpreted to mean that additional hitherto unknown Zn^2+^ uptake mechanisms play a role.

Twenty membrane Mg^2+^ channels/transporters have been proposed (11), including the kinase-coupled channel TRPM6 (12). However, their precise function is surrounded by considerable controversy (11). Recently, we used conditional mutagenesis of *Trpm6* in mice to define its in vivo role (13). We noted that breast-fed *Trpm6*-null pups developed normally. In contrast, weaned *Trpm6*-deficient mice developed severe Mg^2+^ deficiency over a period of about 4 mo due to insufficient Mg^2+^ absorption in the intestine (13). Hence, we anticipate that another intestinal Mg^2+^ uptake channel or transporter is responsible for the normal development of suckling *Trpm6*-deficient pups.

The kinase-channel TRPM7 is the closest homolog of TRPM6 (12) and has been suggested to control cellular Mg^2+^ levels (14–17). Other studies have proposed alternative functions for TRPM7, such as a Ca^2+^ channel involved in cellular signaling (18–20), as an intracellular Zn^2+^ channel regulating cytosolic and vesicular Zn^2+^ levels (21), or even as a H^+^ channel (22). Mice with a constitutive null mutation in *Trpm7* die before embryonic day 6.5–7.5 (17, 23). Tissue-specific mutagenesis of *Trpm7* revealed that TRPM7 is required only before and during organogenesis for reasons that are not fully understood (23–25).

In the present paper, we explored the functional role of TRPM7. By analysis of single-channel characteristics of TRPM7 in lipid bilayers, assessment of TRPM7-deficient cells, and phenotyping of mouse strains with organ-restricted null mutations in *Trpm7*, we define TRPM7 as a central gatekeeper controlling the organismal balance of Zn^2+^, Mg^2+^, and Ca^2+^.

## Results

### *TRPM7*-Deficient HAP1 Cells Display Altered Handling of Zn^2+^ and Mg^2+^.

**Fig. 1.**
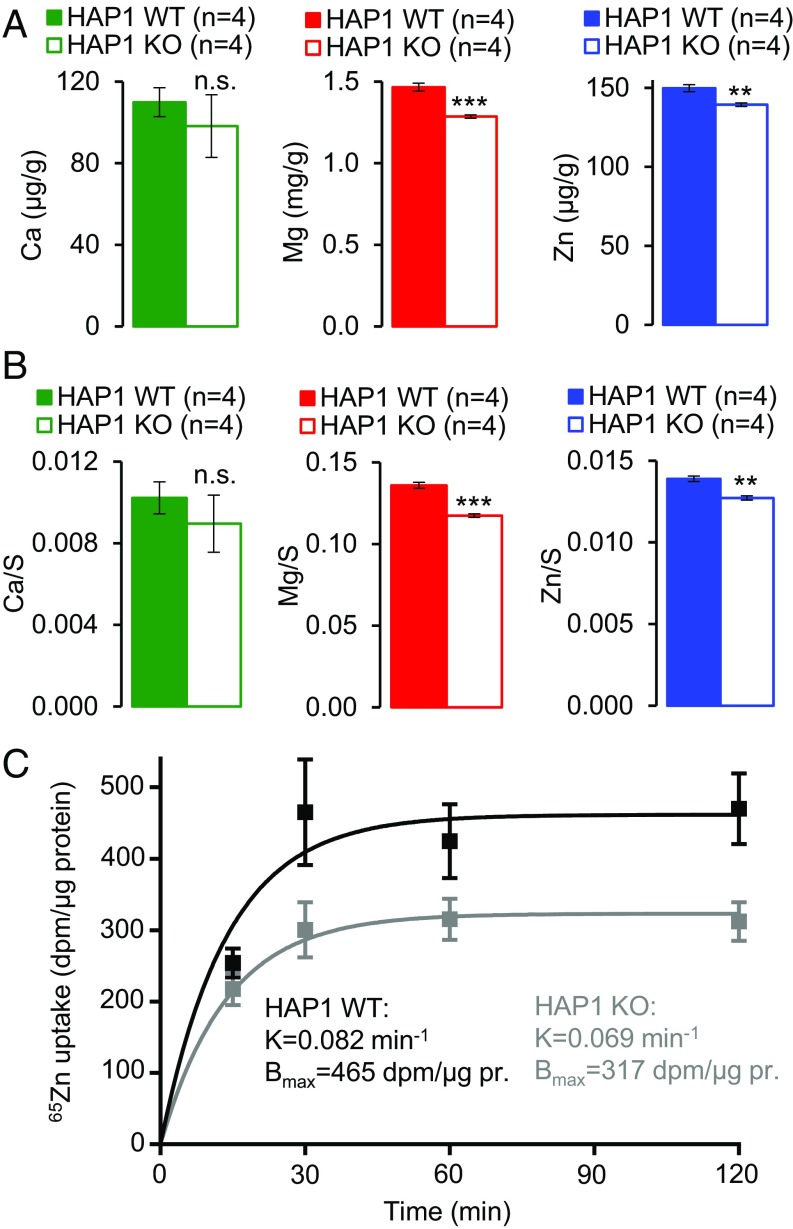
Assessment of human haploid leukemia (HAP1) cells deficient in *TRPM7*. (*A*) Determination of elementary levels of Ca (*Left*), Mg (*Center*), and Zn (*Right*) in wild-type (WT) and *TRPM7*-deficient (KO) HAP1 cells. Dried cell pellets were obtained from WT and KO HAP1 cells cultured in a standard cell culture medium for 24 h and analyzed by ICP-MS. Total elementary contents were normalized to dry pellet weight and represented as mean ± SEM of *n* = 4 independent measurements. (*B*) Total elementary contents of divalent metals obtained in *A* were normalized to total elementary contents of sulfur (S) and represented as mean ± SEM. ***P ≤ 0.001; **P ≤ 0.01; n.s., not significant (Student’s *t* test). (*C*) Uptake of ^65^Zn^2+^ in WT and KO HAP1 cells. Cells were incubated in the presence of 1 mM Mg^2+^, 2 mM Ca^2+^, and 2 µM ^65^Zn^2+^ and time-dependent accumulation of ^65^Zn^2+^ was determined, presented as mean ± SEM for *n* = 6 independent measurements. Datasets were fitted using a one-phase exponential association equation followed by a statistical assessment with the extra sum-of-squares F test.

While the ability of TRPM7 to shape cytosolic Ca^2+^ concentrations has frequently been reported (12), the role of TRPM7 in maintaining cellular Zn^2+^ and Mg^2+^ levels remains incompletely understood. Since most of cellular Zn^2+^ and Mg^2+^ is bound to proteins and metabolites (1, 2), we asked whether TRPM7 is required to maintain the total cellular content of divalent metals. To circumvent the pitfalls imposed by the overexpression of recombinant TRPM7, we took advantage of human haploid leukemia (HAP1) cells that lack endogenous TRPM7 currents due to a frameshift mutation introduced in the *TRPM7* locus (13). Using inductively coupled plasma mass spectrometry (ICP-MS), we found that *TRPM7*-deficient HAP1 cells had significantly reduced intracellular levels of elementary zinc (Zn) and magnesium (Mg) (Fig. 1*A*). In the absence of TRPM7, total cellular elementary calcium (Ca) concentrations trended toward a reduction, but these changes were not statistically significant (Fig. 1*A*). Next, we normalized the levels of elementary Zn, Mg, and Ca to cellular sulfur (a biomarker of the total protein and amino acid content) (Fig. 1*B*), rubidium (as an approximation of potassium levels less prone to environmental contamination; *SI Appendix*, Fig. S1*A*), and phosphorus (as a substitute parameter of total DNA, RNA, and nucleotides; *SI Appendix*, Fig. S1*B*). These analyses revealed that relative levels of Zn and Mg (but not Ca) were significantly reduced in *TRPM7*-deficient HAP1 cells. Consequently, we asked whether the addition of exogenous Zn^2+^ to the cultured medium could circumvent the lack of TRPM7. To this end, we compared Zn levels in control and *TRPM7*-deficient HAP1 cells cultured either in the standard cell culture medium containing ∼3 µM Zn^2+^ or in the medium containing an additional 50 µM ZnSO_4_ (*SI Appendix*, Fig. S1*C*). We found that total content of Zn was significantly increased in both control and *TRPM7*-deficient cells maintained in the Zn^2+^-enriched medium (*SI Appendix*, Fig. S1*C*), indicating that alternative Zn^2+^ uptake mechanisms exist in HAP1 cells. Moreover, we observed that Zn levels were not different in control vs. *TRPM7*-deficient HAP1 cells after Zn^2+^ supplementation (*SI Appendix*, Fig. S1*C*), arguing that differences in Zn^2+^ environment can significantly affect the phenotype of *TRPM7*-deficient cells.

Next, we investigated whether inactivation of native TRPM7 would be sufficient to reduce the uptake of radioactive ^65^Zn^2+^ in resting cells kept in saline containing physiological levels of divalent cations. We incubated wild-type (WT) and *TRPM7*-deficient HAP1 cells in the presence of 1 mM Mg^2+^, 2 mM Ca^2+^, and 2 µM ^65^Zn^2+^ and determined the time-dependent accumulation of ^65^Zn^2+^ (Fig. 1*C*). Datasets were fitted using a one-phase exponential association equation. *TRPM7*-deficient HAP1 cells were characterized by a reduction of ^65^Zn^2+^ uptake over time compared with WT cells (*P* = 0.0005, F test) (Fig. 1*C*). The rate constant (K) was not altered (*P* = 0.72, F test), whereas maximum ^65^Zn^2+^ uptake at equilibrium (B_max_) was significantly reduced in *TRPM7*-deficient cells (*P* = 0.0017, F test). Hence, native TRPM7 regulates the influx of Zn^2+^ in resting cells under physiological conditions.

### The TRPM7 Channel in Planar Lipid Bilayers Is Highly Permeable to Zn^2+^ and Mg^2+^.

**Fig. 2.**
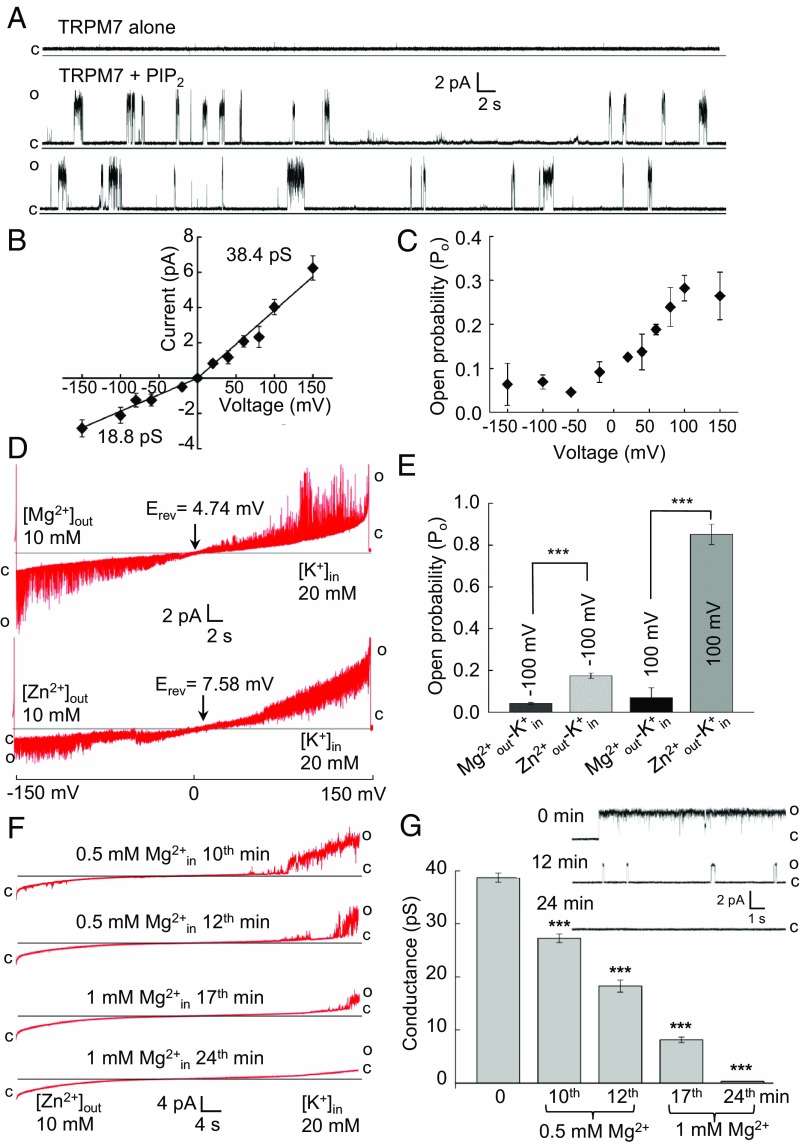
Assessment of TRPM7 channel activity in lipid bilayers. (*A*) Single-channel recordings of TRPM7 at +100 mV in the absence or presence of 5 µM PIP_2_. Representative current traces of TRPM7 were obtained from *n* = 5 independent experiments (*n* = 1,565 events analyzed). The closed and open states of TRPM7 are outlined by c and o, respectively. (*B* and *C*) Voltage sensitivity of the TRPM7 channel as assessed by analysis of mean slope conductance (*B*) and open probability (P_o_) (*C*) of TRPM7 currents measured in the presence of 2.5 µM PIP_2_. Data are presented as mean ± SEM (*n* = 11, *n* = 149,763). Experiments in *A*–*C* were performed under symmetric ionic conditions (*Materials and Methods*). (*D*) Representative current traces of TRPM7 channel activity under Mg^2+^_out_−K^+^_in_ or Zn^2+^_out_−K^+^_in_ biionic conditions in the presence of 2.5 µM PIP_2_ (*n* = 4 for each condition, *n* = 10,521). The voltage-ramp recordings were obtained over a period of 90 s. Reversal potentials (E_rev_) were 4.74 ± 0.46 mV at Mg^2+^_out_−K^+^_in_ (mean ± SEM, *n* = 11) and 7.58 ± 0.74 mV at Zn^2+^_out_−K^+^_in_ conditions (mean ± SEM, *n* = 8). (*E*) P_o_ (mean ± SEM) of TRPM7 calculated for measurements shown in *D*. (*F* and *G*) Effects of Mg^2+^_in_ on TRPM7 currents. (*F*) Representative traces of TRPM7 currents evoked analogously to *A* followed by a sequential application of 0.5 mM and 1 mM Mg^2+^ (*n* = 3). (*G*) Time-dependent suppression of TRPM7 conductance (mean ± SEM, *n* = 15, *n* = 5,642) calculated from the measurements shown in *F*. *G*, *Inset* shows representative traces of single-channel recordings of TRPM7 at +100 mV obtained at 0 min, 12 min, and 24 min. ***P ≤ 0.001 (one-way ANOVA).

Assessments of TRPM7 currents in whole-cell patch-clamp experiments showed that TRPM7 is a constitutively active channel permeable to a variety of divalent cations (14, 26–29). However, as discussed in the aforementioned papers, an accurate comparison of the permeation ratios of divalent cations based on the Goldman–Hodgkin–Katz equation is error prone since TRPM7 currents display a very shallow slope around the reversal potentials (E_rev_) and even small changes in the activity of other channels can affect accurate determination of E_rev_. Therefore, we investigated the ion permeability profile independently by analyzing the single-channel activity of TRPM7 in planar lipid bilayers. This approach has proved instrumental in the biophysical characterization of other TRP channels in a tightly controlled experimental environment (30–32). To this end, we purified recombinant TRPM7, reconstituted the channel in lipid bilayers, and assessed its key functional properties (Fig. 2). In accordance with whole-cell patch-clamp experiments (19), we found that in lipid bilayers the TRPM7 channel is strongly dependent on phosphatidylinositol 4,5-bisphosphate (PIP_2_). Addition of PIP_2_ alone was sufficient to induce channel openings (Fig. 2*A*). As expected (33, 34), analysis of mean slope conductance (Fig. 2*B*) and open probability (P_o_) (Fig. 2*C*) revealed a notable voltage sensitivity of TRPM7. Next, we studied whether the TRPM7 activator naltriben (35) and the TRPM7 inhibitor NS8593 (36) influence channel properties in this system. Naltriben enhanced P_o_ of outwardly directed currents, whereas coapplication of NS8593 inhibited P_o_ of TRPM7 (*SI Appendix*, Fig. S2).

In light of the role of TRPM7 in the regulation of cellular Zn^2+^ and Mg^2+^ (Fig. 1), we investigated the permeability of TRPM7 for Zn^2+^ and Mg^2+^ (Fig. 2*D*). Because Zn^2+^ is poorly soluble above 10 mM, we used external solutions containing 10 mM of the individual divalent cations counterbalanced by an electrogenic solution containing 20 mM K^+^. By this approach, we were able to accurately determine E_rev_ (Fig. 2*D*). Based on the Goldman–Hodgkin–Katz equation, the permeability ratios were P_Zn_/P_K_ = 1.21 ± 0.13 and P_Mg_/P_K_ = 0.94 ± 0.08, indicating that TRPM7 is modestly more permeable to Zn^2+^ than to Mg^2+^.

Importantly, we observed that, in contrast to Zn^2+^, external Mg^2+^ strongly suppressed channel gating of TRPM7, resulting in P_o_ of <0.1 in Mg^2+^_out_−K^+^_in_ settings (Fig. 2*E*). To further investigate the sensitivity of TRPM7 to Mg^2+^, we measured TRPM7 currents in Zn^2+^_out_−K^+^_in_ conditions after sequential application of 0.5 mM and 1 mM Mg^2+^ on the internal aspect of TRPM7 (Fig. 2*F*). Mg^2+^ exposure resulted in a time-dependent suppression of TRPM7 currents (Fig. 2*F*). Further analysis showed that, along with the reduced conductance, P_o_ of TRPM7 was also decreased in the presence of Mg^2+^ at the inner side of the channel (Fig. 2*G*). These results are fully consistent with the concept that Mg^2+^ acts as a negative gating regulator of TRPM7 (14).

Collectively, our results suggest that TRPM7 is able to conduct Zn^2+^ in the presence of physiological levels of Mg^2+^. To investigate the potential physiological relevance of these findings, we studied the phenotypes of several mouse lines carrying mutations in the *Trpm7* locus.

### Inactivation of *Trpm7* in the Kidney Has No Effect on Mineral Homeostasis.

First, we studied whether TRPM7 plays a role in the renal reabsorption of divalent cations using a mouse line carrying a conditional mutation in *Trpm7* (*Trpm7*^*fl*^) (23). In this model, Cre-mediated deletion of the floxed sequence results in a frameshift mutation in *Trpm7* (*Trpm7*^*Δ17*^). Early physiological studies gave rise to the concept that the distal convoluted tubule (DCT) of the kidney determines the final urinary content of Mg^2+^ and Ca^2+^ and, consequently, plays a pivotal role for the whole-body balance of these minerals (37). Therefore, we utilized *Ksp1.3-Cre* transgenic mice, which are widely used for the ablation of floxed alleles in epithelial cells of the collecting duct, the DCT, and the thick ascending limb of Henle (38). *Trpm7*^*fl/fl*^*;Ksp1.3-Cre* (*Trpm7*-kidney KO) animals were obtained at the expected Mendelian frequency and showed no obvious changes in physical appearance compared with control (*Trpm7*^*fl/fl*^) littermates (*SI Appendix*, Table S1). We used in situ hybridization (ISH) to verify the efficient mutagenesis of *Trpm7*. In line with other studies (39–41), we observed that in control kidneys, *Trpm7* was abundantly expressed in tubule segments characterized by apically located cell nuclei, a unique feature of DCT cells (*SI Appendix*, Fig. S3*A*). On the contrary, WT *Trpm7* transcripts were not detectable in tissues of *Trpm7*-kidney KO mice (*SI Appendix*, Fig. S3*A*).

Next, we used ICP-MS to measure mineral levels in serum and urine of 8-wk-old mice. Much to our surprise, conditional *Trpm7* inactivation in the kidney did not entail altered serum concentrations of Zn, Mg, and Ca (*SI Appendix*, Fig. S3*B*). Moreover, mutant mice had normal urinary excretion of divalent cations (*SI Appendix*, Fig. S3*C*). To study whether the lack of TRPM7 was compensated by up-regulation of other genes known to be involved in renal reabsorption of divalent cations, we investigated mRNA levels of *Trpm6*, *Claudin-16*, *ZnT1*, *ZnT2*, and *Trpv5* (1–3) (*SI Appendix*, Fig. S3*D*). However, we did not observe any alterations in mRNA levels of these genes. Hence, the organismal balance of divalent cations is not impaired in *Trpm7* kidney-specific KO mice.

### Deletion of *Trpm7* in Intestinal Epithelial Cells Leads to Early Growth Failure and Death.

**Fig. 3.**
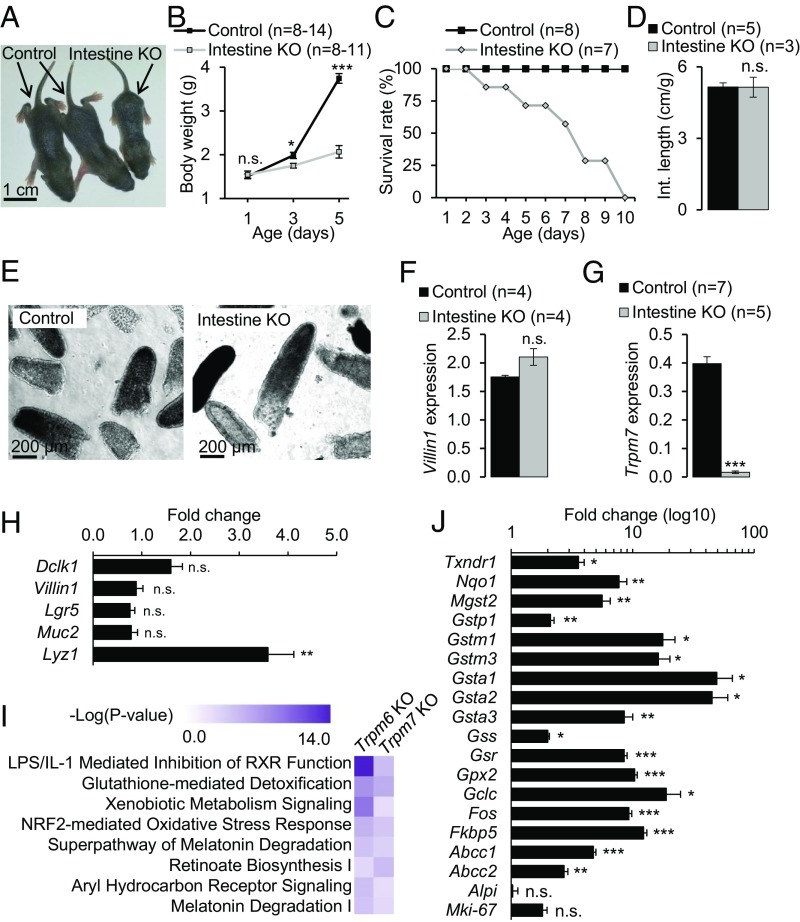
Characterization of mice with intestine-restricted inactivation of *Trpm7*. (*A*–*D*) *Trpm7*^*fl/fl*^ (control) and *Trpm7*^*fl/fl*^*;Villin1-Cre* (intestine KO) littermates were studied for overall physical appearance at P5 (*A*) and growth (*B*), survival rate (*C*), and total length of the intestine normalized to body weight at P5 (*D*). The results in *B* and *D* are represented as mean ± SEM. (*E*–*H*) Assessment of *Villin1-Cre*–mediated deletion of WT *Trpm7* transcripts in the intestine of *Trpm7*^*fl/fl*^*;Villin1-Cre* mice. (*E*) Representative images of purified villi isolated from the whole intestine of P5 littermates. (*F* and *G*) Relative expression levels (mean ± SEM) of *Villin1* (*F*) and *Trpm7* (*G*) in mRNA extracts from villi examined by qPCR using *Hprt* as a reference transcript. (*H*) Relative expression levels of *Dclk1*, *Villin1*, *Lgr5*, *Muc2*, and *Lyz1* in mRNA extracts from the whole duodenum analyzed by qPCR using *Hprt* as a reference transcript. Results are shown as fold change (mean ± SEM) in *Trpm7*^*fl/fl*^*;Villin1-Cre* (*n* = 8) mice vs. *Trpm7*^*fl/fl*^ (*n* = 7) mice. (*I*) Pathways commonly affected in the intestine of *Trpm7*-deficient mice and the liver of *Trpm6*-deficient mice (13). Whole-genome profiling of the transcriptome of villi isolated from P5 *Trpm7*^*fl/fl*^*;Villin1-Cre* (*n* = 3) and *Trpm7*^*fl/fl*^ (*n* = 3) littermates was analyzed using ingenuity pathway analysis (IPA) (Datasets S1 and S2). Next, IPA was used to compare gene networks highly affected in *Trpm7*^*fl/fl*^*;Villin1-Cre* (*Trpm7 KO*) and *Trpm6* null (*Trpm6 KO*) mice (13). (*J*) Relative expression levels of markers of mature and progenitor enterocytes *Alpi* and *Mki-67* and genes representing the IPA glutathione-mediated detoxification and the NRF2-mediated oxidative stress response (Dataset S2) were examined in mRNA extracts from villi of P5 littermates using qPCR, using *Hprt* as a reference transcript (*n* = 3 mice per genotype). The results are shown as fold change (mean ± SEM) in *Trpm7*^*fl/fl*^*;Villin1-Cre* samples vs. *Trpm7*^*fl/fl*^ samples. ***P ≤ 0.001; **P ≤ 0.01; *P ≤ 0.05; n.s., not significant (Student’s *t* test).

Next, we asked whether intestine-restricted inactivation of *Trpm7* would affect the organismal balance of divalent cations. The *Villin1-Cre* transgene allows for the ablation of floxed alleles in enterocytes of the whole intestine (42). Therefore, we compared the phenotypes of *Trpm7*^*fl/fl*^*;Villin1-Cre* (*Trpm7*-intestine KO) with *Trpm7*^*fl/fl*^ (control) littermates (*SI Appendix*, Table S1). *Trpm7*-intestine KO mice were born with the anticipated Mendelian ratio. Overall, the physical appearance of postnatal day 1 (P1) and P2 *Trpm7*-intestine KO pups was indistinguishable from that of control littermates. Macroscopic and histological examination of the small and large intestine of *Trpm7*-intestine KO individuals at P1 did not reveal any abnormalities with regard to gross anatomy and overall mucosal architecture, indicating unaffected intestinal organogenesis (*SI Appendix*, Fig. S4). Rarely, there were discrete histological changes, such as duodenal erosions and increased vacuolization of enterocytes in the basal portion of the jejunum villi resembling the vacuole-rich phenotype of *Trpm7*-deficient megakaryocytes (16) and changes observed in enterocytes of rodents maintained on a Zn^2+^-deficient diet (43) (*SI Appendix*, Fig. S4). During the follow-up period, *Trpm7*-intestine KO individuals were growth retarded and displayed a high mortality. Thus, body weights of P5 *Trpm7*-intestine KO pups were only 45% of controls (Fig. 3 *A* and *B*), only ∼70% of mutants were viable, and all *Trpm7*-intestine KO animals died by P10 (Fig. 3*C*). In contrast, heterozygous littermates (*Trpm7*^*fl/WT*^*;Villin1-Cre*) showed no alterations in survival or growth. Importantly, we observed that the stomachs of P1–10 *Trpm7*-intestine KO pups were filled with milk, indicating normal feeding behavior. Furthermore, we noted that the length of the whole intestine was identical in P5 *Trpm7*-intestine KO pups and control litters (Fig. 3*D*), lending additional credence to the notion that functional deficits rather than developmental abnormalities were responsible for the severe phenotype.

To verify the recombination efficiency of the *Trpm7*^*fl*^ allele in *Trpm7*-intestine KO mice, we isolated villi from the whole intestine of P5 littermates and assessed *Trpm7* mRNA levels by qPCR. We observed no remarkable changes, either in the overall appearance of the villi isolated from mutant mice (Fig. 3*E*) or in the expression levels of *Villin1* (Fig. 3*F*), a marker of differentiated intestinal enterocytes (42). However, WT *Trpm7* transcripts were nearly undetectable in villi from mutant mice (Fig. 3*G*), indicating that *Villin1-Cre* is suitable for efficient mutagenesis of *Trpm7*^*fl*^ in the intestine. The lack of TRPM7 in enterocytes of *Trpm7*-intestine KO mice was further verified using immunofluorescence staining of duodenum sections (*SI Appendix*, Fig. S5). We observed that a TRPM7-specific antibody stained villus epithelial cells in control tissues obtained from 8-wk-old mice and that TRPM7 immunoreactivity was predominantly present on the apical surface of villus epithelial cells. Interestingly, the TRPM7 signal was substantially weaker in control tissues isolated from P5 mice, suggesting that expression levels of TRPM7 are relatively low in intestine of neonatal mice (*SI Appendix*, Fig. S5). In line with the qPCR data (Fig. 3*G*), villus epithelial cells were not stained in tissues of P5 *Trpm7*-intestine KO mice (*SI Appendix*, Fig. S5).

To obtain further insight into tissue homeostasis of *Trpm7*-intestine KO mice, we extracted RNA from the whole duodenum of P5 mutant and control littermates and investigated the expression levels *of Dclk1*, *Villin1*, *Lgr5*, *Lyz1*, and *Muc2*, markers of tuft cells, enterocytes, intestinal stem cells, Paneth cells, and goblet cells, respectively (44). The expression levels of these transcripts were not altered except *Lyz1* was found to be up-regulated (Fig. 3*H*), suggesting that the deletion of TRPM7 did not entail a negative impact on the development of major types of intestinal epithelial cells.

Next, applying an unbiased strategy to examine villus cell homeostasis in *Trpm7*-intestine KO mice, we extracted RNA from villi isolated from the whole intestine of P5 mutant and control littermates and performed genome-wide transcriptome profiling (45) (Dataset S1). Applying a cutoff value of twofold changes with *P* ≤ 0.05 for the false discovery rate (FDR), we identified 455 up- and down-regulated genes in the tissues of *Trpm7*-intestine KO mice (Dataset S2). Intriguingly, the majority of highly affected transcripts code for glutathione *S*-transferases and proteins metabolizing steroids, solute carriers, and cytochrome P450 metabolizing enzymes. These findings were confirmed by qPCR-based assessment of 18 genes involved in the metabolism of glutathione and the oxidative stress response (Fig. 3*J*).

Recently, single-cell RNA sequencing has uncovered that villus enterocytes continuously transdifferentiate as they migrate along the villus axis, reflecting a zonal specialization of enterocytes in absorbing of nutrients and antibacterial defense (44). This study offered a set of transcriptional markers of the bottom, middle, and tip villus enterocytes including *Rpl4*, *Rpl3*, *Cps1*, *Reg3g*, *Reg1*, *Gstm3*, *Enpep*, *Pigr*, *Slc5a1*, *Apoa1*, *Apob*, *Neat1*, *Malat1*, *Ada*, and *Nt5e* (44). We noted that, among these genes, expression of only *Gstm3* and *Enpep* was up-regulated in villi of P5 mutants (*P* ≤ 0.05; Dataset S1). In addition, expression levels of *Alpi* and *Mki67* (markers of mature and progenitor enterocytes, respectively) were unchanged (Fig. 3*J* and Dataset S1). We concluded that the differentiation program of villus enterocytes was not impaired in P5 mutant mice.

We recently reported that inactivation of *Trpm6* is associated with a characteristic induction of gene networks controlling oxidative stress and toxicity responses in the liver (13). Interestingly, oxidative stress has also been linked to TRPM7 function in cultured neurons and other cell types (21, 46). However, it remains unclear whether oxidative stress and toxicity responses can be regarded as a common transcriptional fingerprint of tissues in *Trpm6*- and *Trpm7*-deficient mice. Therefore, we performed ingenuity pathway analysis (IPA) to categorize the 455 affected genes and found that inactivation of *Trpm7* affects gene networks controlling glutathione-mediated detoxification, NRF2-mediated oxidative stress response, cholesterol biosynthesis, and xenobiotic metabolism (Dataset S2). We noted (Fig. 3*I*) that these pathways were also specifically affected in livers of *Trpm6* null mice (13), suggesting that the observed transcriptional alterations do represent a generalized genetic fingerprint evoked by perturbed Mg^2+^ homeostasis. However, a contribution of abnormal Zn^2+^ balance in eliciting such responses is also well documented in the literature (47) and may contribute to the transcriptional profile in *Trpm7*-deficent mice.

Taken together, our experiments suggest that conditional mutagenesis of *Trpm7* in intestinal enterocytes did not affect embryonic morphogenesis of the gut, but rather engenders postnatal metabolic deficits presumably associated with oxidative stress and disrupted Mg^2+^ and/or Zn^2+^ homeostasis.

### Perturbation of Divalent Cation Homeostasis in Mice Lacking Intestinal *Trpm7*.

**Fig. 4.**
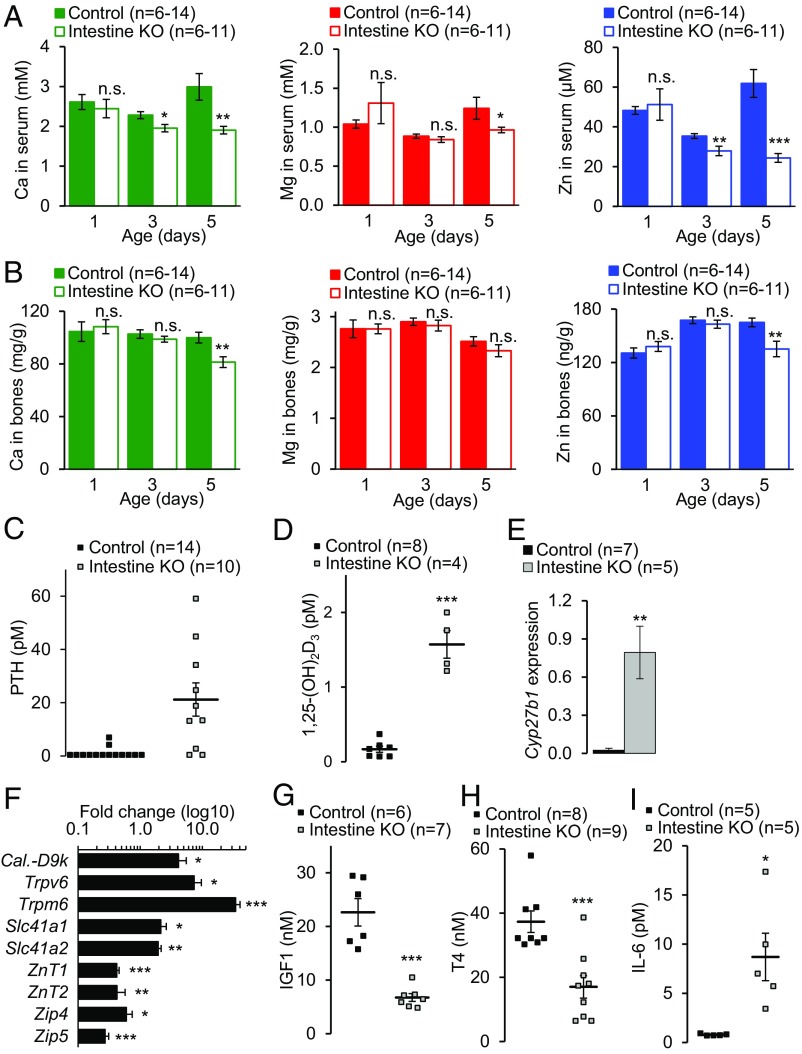
Analysis of Ca^2+^, Mg^2+^, and Zn^2+^ homeostasis in mice with intestine-restricted inactivation of *Trpm7*. (*A* and *B*) ICP-MS analysis of elementary levels of Ca (*Left*), Mg (*Center*), and Zn (*Right*) in serum (*A*) and bones (right tibia) (*B*) of P1, P3, and P5 *Trpm7*^*fl/fl*^ (control) and *Trpm7*^*fl/fl*^*;Villin1-Cre* (intestine KO) littermates (*n*, number of mice examined per data point). In *B* total elementary contents were normalized to dry bone weights (mean ± SEM); n, number of mice per genotype. (*C* and *D*) P5 littermates were examined for circulating levels of PTH (*C*) and 1,25-(OH)_2_D_3_ (*D*) (mean ± SEM). Note that only two control samples contained PTH levels in the range of ELISA sensitivity, and statistical analysis could not be conducted. (*E*) Relative expression levels (mean ± SEM) of *Cyp27b1* in mRNA extracts from whole kidneys of P5 littermates were studied using qPCR and *Ywhaz* as a reference transcript. (*F*) Relative expression levels of indicated transcripts were investigated in mRNA extracts from whole duodenums of P5 littermates using qPCR and *Hprt* as a reference transcript. Results are calculated as fold change (mean ± SEM) in *Trpm7*^*fl/fl*^*;Villin1-Cre* (*n* = 8–12) mice vs. *Trpm7*^*fl/fl*^ (*n* = 7–12) mice. (*G*–*I*) P5 littermates were assessed for circulating levels of IGF1 (*G*), T4 (*H*), and IL-6 (*I*) (mean ± SEM). ***P ≤ 0.001; **P ≤ 0.01; *P ≤ 0.05; n.s., not significant (Student’s *t* test); *n*, number of mice examined.

Based on previous mechanistic studies on the specific contribution of TRPM6 to TRPM7 function in epithelial cells (13), we hypothesized that the disruption of TRPM7 in intestinal enterocytes would result in insufficient uptake of divalent cations. Therefore, using ICP-MS we analyzed levels of metals in serum and bones (right tibia) of mice before (P1) or at the onset (P3, P5) of macroscopic phenotypes displayed by *Trpm7*-intestine KO pups (Fig. 3 *A*–*C*). As expected, we found no changes in concentrations of monovalent (K and Na) (*SI Appendix*, Fig. S6 *A* and *B*) and divalent (Zn, Mg, and Ca) cations in P1 *Trpm7*-intestine KO individuals, suggesting that newborn *Trpm7*-intestine KO mice had normal mineral homeostasis (Fig. 4 *A* and *B*). However, during the follow-up period *Trpm7*-intestine KO mice developed a gradual deficiency of divalent cations. Specifically, we observed that serum levels of Mg were unaltered in P3 *Trpm7*-intestine KO pups, but were only 78% of control values at P5 (Fig. 4*A*). However, Mg contents of bones of P3 and P5 *Trpm7*-intestine KO mice were normal, suggesting that mutant mice developed a rather modest Mg^2+^ deficiency at P5 (Fig. 4*B*). In contrast, serum Ca concentrations were significantly reduced in *Trpm7*-intestine KO mice already at P3 (86% of control values) and further dropped to 64% at P5 (Fig. 4*A*). Consistently, P5 *Trpm7*-intestine KO mice displayed depletion of Ca in bones (Fig. 4*B*). Remarkably, the organismal Zn balance was most strongly affected in *Trpm7*-intestine KO mice. Thus, serum Zn concentrations in *Trpm7*-deficient mice were only 79% and 39% of control values at P3 and P5, respectively (Fig. 4*A*). In bones, Zn levels were not altered in P3 *Trpm7*-intestine KO mice, but substantially decreased in P5 mutants (Fig. 4*B*). Importantly, the concentrations of monovalent cations were unaltered in serum and in bones of P3 and P5 *Trpm7*-intestine KO mice (except an elevation of Na levels in bones of P5 mutants; *SI Appendix*, Fig. S6*B*), suggesting that the deletion of intestinal *Trpm7* caused a specific defect in the uptake of divalent cations.

Next, we asked whether the deficiency of divalent cations would induce an adaptive organismal response in *Trpm7*-intestine KO mice. It is well established that low circulating levels of Ca^2+^ promote the secretion of parathyroid hormone (PTH) and the formation of calcitriol [1,25-(OH)_2_D_3_] (3). Remarkably, we observed an 18.2-fold increase in the levels of PTH (Fig. 4*C*) and a 9.4-fold elevation of 1,25-(OH)_2_D_3_ concentrations (Fig. 4*D*) in the serum of *Trpm7*-intestine KO mice. Organismal Ca^2+^ deficiency triggers the renal production of 1,25-(OH)_2_D_3_ through up-regulation of 25-hydroxyvitamin D_3_ 1-alpha-hydroxylase (*Cyp27b1*) (3). Consequently, in kidneys of P5 *Trpm7*-intestine KO mice the expression of *Cyp27b1* was up-regulated by a factor of 32.4 (Fig. 4*E*). The 1,25-(OH)_2_D_3_ promotes the intestinal uptake of Ca^2+^ by up-regulating the expression of the Ca^2+^-selective TRPV6 channel and the cytosolic Ca^2+^ carrier Calbindin-D9k (3). Assessment of total RNA isolated from whole duodenum confirmed that the expression of *Trpv6* and *Calbindin-D9k* was up-regulated 7.4- and 4.1-fold, respectively, in P5 *Trpm7*-intestine KO mice (Fig. 4*F*). Thus, *Trpm7*-intestine KO mice develop a severe organismal Ca^2+^ deficit.

In contrast to the situation with Ca^2+^, specific adaptive responses to organismal Mg^2+^ or Zn^2+^ deficiency remain poorly understood. Previously, we showed that Mg^2+^ deficiency in *Trpm6* null mice was associated with a growth restriction and an impairment of the growth hormone (GH)/insulin-like growth factor 1 (IGF1) axis (13). Zn^2+^ deficiency was found to be associated with reduced circulating levels of IGF1 (2). In accordance with these observations, serum IGF1 concentrations were also substantially lower in P5 *Trpm7*-intestine KO mice (Fig. 4*G*), suggesting that alterations in the somatotropic axis suppress the postnatal growth of mutant mice. In addition, we observed that *Trpm6* mRNA levels were up-regulated 30.0-fold (Fig. 4*F*). Two putative Mg^2+^ transporters, *Slc41a1* and *Slc41a2*, were also significantly up-regulated (Fig. 4*F*). It has been suggested that abnormal Zn^2+^ homeostasis is intertwined with thyroid gland function (48) and triggers inflammation (49). We therefore investigated serum levels of T_4_ and proinflammatory cytokine IL-6 in P5 littermates and found that *Trpm7*-intestine KO individuals developed severe hypothyroidism (Fig. 4*H*) and had high circulating levels of IL-6 (Fig. 4*I*). Next, we investigated whether the expression of known intestinal Zn^2+^ transporters was affected in the duodenum of P5 *Trpm7*-intestine KO mice. mRNA levels of *ZnT1*, *ZnT2*, *Zip4*, and *Zip5* were significantly down-regulated in mutant pups (Fig. 4*F*).

Early reports from autopsies of subjects with AE syndrome revealed absence of the thymus, a lack of germinal centers in the spleen, and pneumonia or sepsis as likely causes of the death of patients (50–52). Follow-up clinical studies and experiments with animal models confirmed that Zn^2+^ deficiency leads to systemic immune deficiency (2, 49). Therefore, we examined the macroscopic and histological appearance of thymus and spleen in 10 control and 9 *Trpm7*-intestine KO P5 mice (*SI Appendix*, Fig. S7). Notably, two mutant mice were athymic, while the remaining *Trpm7*-intestine KO mice contained only rudimentary organs (*SI Appendix*, Fig. S7 *A* and *B*). Spleens were present in all mutant mice, but the size of the organs was substantially reduced (*SI Appendix*, Fig. S7 *D* and *E*). Histological analysis revealed that *Trpm7*-deficient mice develop remarkable abnormalities in the morphology of lymphoid organs (*SI Appendix*, Fig. S7 *C* and *F*). Thus, the medulla region was not detectable in the thymus of mutant mice. In the spleen of *Trpm7*-deficient mice, the white pulp microarchitecture was not visible. These findings support the idea that, analogously to patients with AE, *Trpm7*-intestine KO mice develop an immune deficiency incompatible with organismal survival.

Taken together, we concluded that the epithelial transport of Zn^2+^, Mg^2+^, and Ca^2+^ is profoundly dysregulated in *Trpm7*-intestine KO mice, triggering a strong organismal response.

### Mice Carrying a Global Kinase-Dead Mutation in *Trpm7* Show Unaltered Organismal Balance of Divalent Cations.

As TRPM7 comprises a channel segment linked to a protein kinase, we asked whether the lack of kinase activity of TRPM7 may play a role in the deficiency of divalent cations in *Trpm7*-intestine KO mice. Recently, a mouse line carrying a global “kinase-dead” K1646R point mutation (*Trpm7*^*R*^) abrogating the catalytic activity of the TRPM7 kinase was generated (53). To this end, we examined the concentrations of divalent metals in serum and bones of 8- to 10-wk-old *Trpm7*^*WT/WT*^ and *Trpm7*^*R/R*^ littermates (*SI Appendix*, Table S1). *Trpm7*^*R/R*^ mice showed unaltered elementary levels of Mg and Zn in serum and bones (*SI Appendix*, Fig. S8). Interestingly, circulating levels of Ca were modestly elevated in *Trpm7*^*R/R*^ mice, whereas the Ca contents of bone were not changed (*SI Appendix*, Fig. S8). These results suggest that the lack of TRPM7 channel, but not catalytic activity of the kinase, primarily triggers the phenotype of *Trpm7*-intestine KO mice. However, at present we cannot exclude that the TRPM7 kinase might contribute to divalent cation homeostasis in other tissues and that the intestinal TRPM6 kinase may compensate for the lack of TRPM7 kinase.

### Dietary Zn^2+^ and Mg^2+^ Fortifications Extend the Lifespan of the Mice Lacking Intestinal TRPM7.

**Fig. 5.**
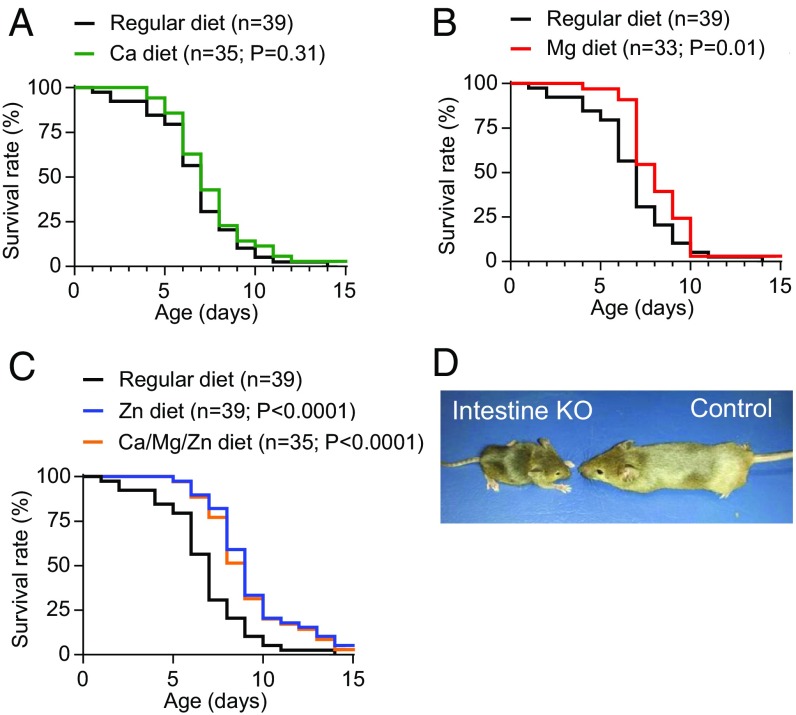
Effects of Ca^2+^, Mg^2+^, and Zn^2+^ dietary treatments on survival of mice with intestine-restricted inactivation of *Trpm7*. (*A*–*C*) Kaplan–Meier survival distributions of *Trpm7*^*fl/fl*^*;Villin1-Cre* mice maintained on a regular diet vs. Ca- (*A*), Mg- (*B*), and Zn-supplemented and combined Ca/Mg/Zn-supplemented mice (*C*) as outlined in *SI Appendix*, Table S2. The indicated *P* values were calculated by comparison of each supplemented group vs. the group maintained at regular diet using a log-rank test; *n*, number of mice examined. (*D*) Representative images of a surviving P33 *Trpm7*^*fl/fl*^*;Villin1-Cre* (intestine KO) mouse and a corresponding *Trpm7*^*fl/fl*^ (control) littermate maintained at an enriched Zn^2+^ diet.

We investigated whether chronic dietary Zn^2+^, Mg^2+^, and Ca^2+^ supplementation of mothers during pregnancy and breastfeeding may ameliorate the high mortality of newborn *Trpm7*-intestine KO mice. To this end, we compared the lifespans of *Trpm7*-intestine KO mice produced by five groups of breeding couples maintained under regular conditions or on Zn^2+^-, Mg^2+^-, and Ca^2+^-rich diets (*SI Appendix*, Table S2). None of the *Trpm7*-intestine KO individuals from mothers fed a normal control diet survived past P14, and those from mothers on a diet supplemented with Ca^2+^ also did not show a significantly extended lifespan (Fig. 5*A*). However, we found that Mg^2+^ supplementation was beneficial for postnatal survival of *Trpm7*-intestine KO offspring (Fig. 5*B*). The onset of mortality in the latter group was substantially delayed, and one mutant individual was viable at weaning. Remarkably, Zn^2+^ supplementation gave rise to a significant rightward shift in the survival curve of *Trpm7*-intestine KO individuals (Fig. 5*C*). In addition, administration of a Zn^2+^-rich diet substantially affected the early mortality of mutants: Only 18% of pups died at P7 in the Zn^2+^-supplemented group vs. 69% in the untreated group. Importantly, two *Trpm7*-intestine KO individuals of the Zn^2+^-treated group survived to weaning (Fig. 5 *C* and *D*). Thus, nutritional Zn^2+^ fortification, and to a lesser extent Mg^2+^ supplementation, significantly extended the lifespan of mice lacking intestinal TRPM7.

Finally, we studied how a combined diet containing increased contents of Zn^2+^, Mg^2+^, and Ca^2+^ can affect mortality of *Trpm7*-intestine KO mice (Fig. 5*C*). We found that the survival curve of the group maintained on the combined diet mirrored that of the Zn^2+^-treated animals and that one mutant mouse survived to weaning. These results support the idea that Zn^2+^ deficiency primarily underlies the mortality of *Trpm7*-intestine KO mice and that additional dietary Mg^2+^ and Ca^2+^ could not circumvent this outcome in our experimental settings.

## Discussion

We demonstrate that ablation of TRPM7 in intestinal enterocytes is sufficient to evoke a striking organismal deficiency in Zn^2+^, Mg^2+^, and Ca^2+^, which is incompatible with early postnatal life of mutant mice and considerably more severe than that of previously reported mouse strains carrying loss-of-function mutations in other known Zn^2+^, Mg^2+^, or Ca^2+^ channels (discussed below). Hence, the intestinal TRPM7 channel functions as a master regulator of the organismal Zn^2+^, Mg^2+^, and Ca^2+^ balance. Herein, we offer a paradigm with the potential to resolve the seeming paradox of TRPM7 functioning as a Ca^2+^ or Mg^2+^ or Zn^2+^ permeable ion channel under physiological conditions. The significance of our observations is a unified view that places TRPM7 in a central position as a common gatekeeper regulating the nutritional uptake of several essential divalent metals and not specific to only one.

Electrophysiological patch-clamp experiments have suggested that TRPM7 is a constitutively active channel highly permeable to divalent cations (14, 26–29). However, these biophysical assessments of TRPM7 were performed using cells exposed to extracellular solutions with high levels of individual cations (typically 10–20 mM or even isotonic solutions) and the complete removal of intracellular divalent cations. Such settings are very artificial and, therefore, difficult to extend to endogenously occurring TRPM7 currents under physiological conditions. In particular, it has remained unclear whether the channel would be capable of conducting Zn^2+^ ions under physiological conditions, i.e., in the presence of a >100-fold excess of external Ca^2+^ and Mg^2+^. To address this question, we investigated the ability of endogenous TRPM7 to regulate the uptake of ^65^Zn^2+^ and to impact the total elementary Zn content in resting HAP1 cells incubated in the presence of physiological levels of extracellular Zn^2+^, Ca^2+^, and Mg^2+^. These experiments demonstrated that the TRPM7 channel plays a nonredundant role in the cellular uptake of Zn^2+^. Furthermore, in accordance with previous studies (13, 15, 17), we observed that the lack of TRPM7 is associated with a reduced cellular content of elementary Mg. These results are in line with the concept that the ubiquitously expressed TRPM7 channel is responsible for the bulk uptake of divalent metals into cells. Accordingly, a particular cellular response to the ablation of TRPM7 currents will likely depend on (*i*) the actual metabolic demand of the cell and (*ii*) the complement of Zn^2+^, Mg^2+^, and Ca^2+^ channels/transporters within the cell. For instance, insulin production in pancreatic beta cells and immune responses of myeloid and lymphoid cells are highly dependent on Zn^2+^ (2) and, consequently, ablation of TRPM7 may primarily affect Zn^2+^-dependent processes in these cells. Proliferation of cancer cells depend on TRPM7-mediated Mg^2+^ influx since cellular levels of Mg·ATP and Mg-bound metabolites have to be duplicated during cell division (12), and so the Mg^2+^-dependent process would likely be primarily affected by the ablation of TRPM7 in such cells. Finally, such a concept is not mutually exclusive with the idea that TRPM7 is capable of inducing transient rises of cytosolic Ca^2+^ to trigger signaling events, for instance, in oocytes, cardiac myocytes, and neurons (12).

In this study, we were able to reconstitute the TRPM7 channel in lipid bilayers. Analysis of TRPM7 single-channel activity highlighted crucial roles of PIP_2_ and Mg^2+^ for channel gating (14, 19). These results lend support to the idea that cytosolic Mg^2+^ may serve as a “fasting” feedback mechanism, which links energy metabolism and cell cycle to the opening of the TRPM7 channel (14, 15). By reconstituting TRPM7 in a well-controlled experimental environment we observed that the TRPM7 channel is slightly more selective for Zn^2+^ compared with Mg^2+^ and able to conduct Zn^2+^ in the presence of physiological levels of cytosolic free Mg^2+^, mirroring the phenotype of TRPM7-deficient cells and animals.

A key finding of this study is that the preference of the TRPM7 channel pore for Zn^2+^ appears to correlate with the in vivo role of TRPM7. Mice lacking intestinal TRPM7 exhibit severe Zn^2+^ deficiency, as serum Zn^2+^ concentrations were normal in neonatal mutants at P1, but dropped to 39% of control values by P5, accompanied by a significant loss of Zn^2+^ in bones. Importantly, excess Zn^2+^ in the drinking water of nursing dams significantly improved the survival of *Trpm7*-intestine KO mice. Since TRPM7 kinase can potentially phosphorylate other Zn^2+^ channels and transporters, and thus may potentially modify the intestinal uptake of Zn^2+^, we investigated a kinase-dead *Trpm7*^*R/R*^ mouse line and found that the catalytic activity of the TRPM7 kinase is not required for the maintenance of Zn^2+^ homeostasis.

Compared with existing animal models with defects in ZnT or Zip proteins, the early onset and very fast development of Zn^2+^ deficiency in mice lacking intestinal TRPM7 appear to reflect a pronounced defect in Zn^2+^ reabsorption. For instance, mice with spontaneous mutations in *ZnT4*, referred to as lethal milk (*lm*) mutations, are characterized by a ∼35% reduction of Zn^2+^ content in milk of *lm/lm* dams, sufficient to entail 100% mortality of suckled pups within 2 wk unless foster nurtured by WT dams (4, 54). However, after weaning, *ZnT4*-deficient mice are asymptomatic. As already mentioned, mutations in the human *ZIP4* gene cause AE syndrome associated with a defect in the intestinal uptake of Zn^2+^ (4, 55). Among other symptoms, untreated patients with AE displayed growth retardation, anorexia, immunodeficiency associated with fungal and bacterial infections, and mortality (4, 55). Similar to that in patients with AE, an intestine-restricted inactivation of *Zip4* in mice did not exert deleterious effects on breast-fed pups (10). However, weaned *Zip4*-deficient mice displayed reduced Zn^2+^ levels in internal organs, growth retardation, and death unless mutants were supplemented with excess Zn^2+^ (10). Unfortunately, serum and bone Zn^2+^ levels were not reported for *Zip4*-deficient mice (10). We noted that *Trpm7*-intestine KO mice reliably phenocopied the aforementioned pathophysiological alterations, further supporting the idea that TRPM7 is a vital component of the intestinal uptake machinery of Zn^2+^. Our results suggest also that organismal imbalance of Zn^2+^ damages immune tissues of *Trpm7*-intestine KO mice, resulting in the high mortality of animals, in analogy to subjects with AE. While the role of TRPM7 in mineral absorption is critical for the survival of postnatal mice, we cannot rule out the possibility that TRPM7 may not be equally essential in the adult organism, because the nutritional demand for Zn^2+^ may be lower in adult than in fast-growing postnatal animals. Therefore, conditional inactivation of *Trpm7* in adult mice may have a less severe impact on the survival and the phenotype of mutant animals.

The Ca^2+^-selective TRPV6 channel was proposed to function as a major mechanism for transcellular Ca^2+^ transport in the intestine (7). However, *Trpv6* null mice displayed normal serum Ca^2+^ levels (7). Furthermore, two other studies found no effect of *Trpv6* null mutation to intestinal Ca^2+^ absorption (8, 9), suggesting that the TRPV6 channel is functionally redundant. In this paper, we show that mice lacking intestinal TRPM7 develop severe Ca^2+^ deficiency, already evident at early postnatal stages. Thus, mutant P5 pups displayed a strong decrease in Ca^2+^ concentrations in both serum and bones, accompanied by profound increases in circulating levels of PTH and 1,25-(OH)_2_D_3_ and in expression levels of *Cyp27b1*, *Trpv6*, and *Calbindin-D9k*. These findings suggest that TRPM7, rather than TRPV6, represents the key mechanism of intestinal Ca^2+^ reabsorption.

Recently, we demonstrated that weaned mice with a global *Trpm6* null mutation develop a severe Mg^2+^ deficiency due to a defect in intestinal Mg^2+^ uptake, which leads to alterations in the somatotropic axis and growth restriction of 8- to 12-wk-old mice (13). Furthermore, intestine-restricted inactivation of *Trpm6* triggered a more moderate Mg^2+^ deficiency, suggesting that WT kidneys were not fully capable of compensating for the lack of intestinal TRPM6 (13). In addition, we and others have demonstrated that the specific functional contribution of TRPM6 to Mg^2+^ absorption is to relieve the heterogenic TRPM6/M7 channel from inhibition by intracellular Mg·ATP (13, 28, 40, 56). In line with this model, we show here that intestine-restricted inactivation of *Trpm7* is sufficient to induce systemic Mg^2+^ deprivation in early postnatal life in conjunction with reduced circulating levels of IGF1, a 30-fold up-regulation of intestinal *Trpm6* mRNA levels, and induction of genes associated with oxidative stress, a known consequence of Mg^2+^ deficiency (1). Finally, we observed that Mg^2+^ supplementation was beneficial for the survival of *Trpm7*-intestine KO mice.

Overall, our findings impose a critical revision of the current view of intestinal mineral uptake in particular and organismal mineral homeostasis in general. Our results indicate that whole-body balance of Zn^2+^, Mg^2+^, and Ca^2+^ critically depends on a common gatekeeper, the intestinal TRPM7 channel, whose action is likely fine-tuned by downstream activity of cation-specific transporters/channels. Accordingly, a systematic assessment of TRPM7 function in human syndromes characterized by divalent cation deficiency should be enlightening in this regard.

## Materials and Methods

### Examination of Human HAP1 Cells.

Experiments with cells were approved by the local councils (permits AZ: 55.1-8791-14.718 and 55.2-1-54-2532-180-2016 from Government of Oberbayern). WT and *TRPM7*-deficient HAP1 cells were described previously (13). Cellular content of main elements in dry pellets of HAP1 cells was determined by ICP-MS as reported before (13) with a few modifications (*SI Appendix*, *SI Materials and Methods*). Determination of ^65^Zn^2+^ uptake in HAP1 cells is described in *SI Appendix*, *SI Materials and Methods*.

### Planar Lipid Bilayer Measurements.

Expression of mouse TRPM7 with a C-terminal Myc tag from the pcDNA3.1/V5-His TA-TOPO vector (TRPM7-Myc) was reported previously (28, 40). Human embryonic kidney 293 (HEK293) cells were maintained in Eagle’s minimum essential medium (ATCC) supplemented with 10% FBS and 1% penicillin/streptomycin (Lonza) at 37 °C in 5% CO_2_. TRPM7-Myc was transiently transfected in HEK293 cells using Effectene reagent (Qiagen). Purification of TRPM7-Myc was performed as described previously (31, 32) with a few modifications. HEK293 cells expressing TRPM7-Myc were washed two times with PBS (Thermo Fisher Scientific), mechanically disaggregated in PBS, and collected by centrifugation (2,500 × *g*, 5 min). The cell pellet was resuspended in a NaCl-based (NCB) buffer containing 500 mM NaCl, 50 mM NaH_2_PO_4_, and 20 mM Hepes (pH 7.5) supplemented with 10% glycerol, 1 mM phenylmethane sulfonyl fluoride (PMSF) (Roche Biochemical Reagents), and 5 mM β-mercaptoethanol. The cells were subjected to two freeze-thaw cycles and were centrifuged at 40,000 × *g* for 2.5 h. The obtained pellet was solubilized in the NCB buffer containing a protease inhibitor mixture (Roche Biochemical Reagents), 0.1% Nonidet P-40 (Roche Diagnostics), and 0.5% dodecylmaltoside (EMD Biosciences) overnight at 4 °C on a rotator. The lysate was clarified by centrifugation for 1 h at 40,000 × *g* and the supernatant was used for immunoprecipitation of TRPM7-Myc with an anti-Myc antibody (M5546; Sigma-Aldrich) and protein-A/G–conjugated magnetic beads (PI88803; Thermo Fisher Scientific) according to the manufacturer’s protocol. Briefly, the magnetic beads with the bound TRPM7-Myc were washed five times with the NCB buffer followed by an additional washing step with the NCB buffer containing 0.1% Nonidet P-40 and 0.03% lauryl maltose neopentyl glycol (LMNG). TRPM7-Myc was eluted using the NCB buffer containing 0.1% Nonidet P-40, 0.03% LMNG, and 150 µg/mL of Myc-peptide (Thermo Fisher Scientific). All purification steps were performed at 4 °C. Isolation of TRPM7-Myc was verified by 10% polyacrylamide gel electrophoresis (BioRad) and a silver-stain detection of ∼212 kDa TRPM7-Myc.

Planar lipid bilayer measurements were performed as previously described (30–32). Briefly, planar lipid bilayers were formed using 1 mg/mL of synthetic 1-palmitoyl-2-oleoyl-glycero-3-phosphocholine (POPC) and 1-palmitoyl-2-oleoyl-glycero-3-phosphoethanolamine (POPE) (Avanti Polar Lipids) in n-decane (Sigma-Aldrich) with 3:1 POPC/POPE ratio. The POPC-POPE mixture was used to paint a bilayer in an aperture of ∼150 µm diameter in a Delrin cup (Warner Instruments) between symmetric solutions containing 150 mM KCl, 20 μM MgCl_2_, 1 μM CaCl_2_, and 20 mM Hepes (pH 7.2). To perform experiments under biionic conditions, external solutions contained 10 mM of individual divalent cations (Zn^2+^ is poorly soluble above 10 mM at pH 7.2), which was counterbalanced by an internal solution comprising 20 mM K^+^. In particular, we used either 10 mM MgCl_2_/20 mM KCl (Mg^2+^_out_−K^+^_in_ conditions) or 10 mM ZnCl_2_/20 mM KCl (Zn^2+^_out_−K^+^_in_ conditions) in 20 mM Hepes (pH 7.2). All salts were ultrapure (>99%) (Sigma-Aldrich). Unless stated differently, all experiments were performed in the presence of 2.5 μM 1-(1,2R-dioctanoylphosphatidyl)inositol 4,5-bisphosphate, trisodium salt (DiC8-PIP_2_; Cayman Chemical), which was added to both compartments. Bilayer capacitances were in the range of 50–75 pF. Before incorporation to the bilayer, TRPM7-detergent micellar solution (1–2 ng/mL) was added to the POPC-POPE mixture at a ratio of 1:10. The TRPM7-containing micelles were applied to the bilayers by a painting approach. Currents were recorded using a patch-clamp amplifier (Axopatch 200B; Molecular Devices-Axon). The *trans* solution (voltage command side) was connected to a CV 201A head-stage input. The *cis* solution was held at virtual ground via a pair of matched Ag-AgCl electrodes. Background currents measured in the voltage-clamped bilayers in the absence of TRPM7 (<1 pS) were filtered at the amplifier output (low pass, −3 dB at 10 kHz, eight-pole Bessel response). Data were filtered at 100 Hz through an eight-pole Bessel filter (950 TAF; Frequency Devices) and digitized at 1 kHz with an analog-to-digital converter (Digidata 1322A; Molecular Devices) controlled by pClamp10.3 software (Molecular Devices). Single-channel conductance events, all-points histograms, open probability (Po), and other parameters were analyzed using Clampfit 10.3 software (Molecular Devices). Experiments were performed at ∼23 °C.

Permeability ratios were determined using the Goldman–Hodgkin–Katz equation (57) for the conditions when Mg^2+^ (Zn^2+^) is the only permeant cation in the external bath solution and K^+^ is the only permeant cation in the internal solution,

$${\text{E}}_{\text{rev}}=\frac{\text{RT}}{2\text{F}}\text{ln}\frac{4{\text{P}}_{\text{x}}{\left[{\text{a}}_{\text{x}}\right]}_{\text{out}}}{{\text{P}}_{\text{K}}{\left[{\text{a}}_{\text{k}}\right]}_{\text{in}}},$$

where *E*_rev_ is the reversal potential; *P* is the permeability of the ion; x is the divalent ion (Mg^2+^ or Zn^2+^); a is the activity of the ion, equal to ion concentration multiplied by activity coefficient of the ion; R is the universal gas constant; T is the temperature, and F is Faraday’s constant. A one-Way ANOVA statistical analysis of variance and then a Fisher’s least-significant difference test were performed using Origin version 9.0 software (Microcal Software Inc.). Significance was accepted at *P* ≤ 0.05.

### Mouse Strains, Housing of Animals, and Dietary Supplementations.

Experiments with mice were performed in accordance with the European Union Animal Welfare Act and were approved by the local councils on animal care (permit 55.2-1-54-2532-180-2016 from Government of Oberbayern). A mouse line carrying a conditional mutation in *Trpm7* (*Trpm7*^*fl/fl*^ mice, 129S6/SvEvTac) was kindly provided by David Clapham, Janelia Farm Research Campus, Howard Hughes Medical Institute, Ashburn, VA (23). Mice expressing Cre recombinase under the control of the mouse *Kidney-specific promoter* (*Ksp1.3-Cre* mice, C57BL/6J) and the *Villin1* promoter (*Villin1-Cre* mice, C57BL/6J) were obtained from Jackson laboratory (stock nos. 012237 and 004586, respectively). To conditionally inactivate *Trpm7*, *Ksp1.3-Cre* and *Villin1-Cre* mice were crossed with *Trpm7*^*fl/fl*^ mice (*SI Appendix*, Table S1). Mice with a global kinase-dead point mutation in *Trpm7* (*Trpm7*^*R/R*^ mice) were reported earlier (53). Mice were maintained in individually ventilated polycarbonate cages (IVC System; Tecniplast). Cages were changed weekly and were on a 12-h light/dark cycle with artificial lighting. Temperature and relative humidity were 22 ± 1 °C and 50 ± 5%, respectively. Breeding animals were maintained on a multigrain chow (Ssniff M-Z; Ssniff GmbH) and drinking water containing 20.4 mg/L Mg^2+^, 79.6 mg/L Ca^2+^, and <0.2 mg/L Zn^2+^ (ad libitum). Litters were weaned at 3 wk of age and genotyped and desired littermates were housed in cages as described above except that a maintenance chow (Ssniff R/M-H; Ssniff GmbH) was used.

For supplementation experiments with *Trpm7*^*fl/fl*^*;Villin1-Cre* mice, breeding couples and the corresponding weaned offspring were maintained ad libitum on five dietary regimes (*SI Appendix*, Table S2). Deionized drinking water (MembraPure, resistance >18 MΩ, total organic carbon <20 ppb) was administered to all groups. Mice administered the standard maintenance chow Ssniff R/M-H (Ssniff GmbH) containing 0.22% Mg^2+^, 1.00% Ca^2+^, and 0.089% Zn^2+^ were referred to as a group maintained on a “regular diet.” A “high-Mg diet” was designed using the maintenance chow Ssniff R/M-H with additional 0.53% Mg^2+^ using MgO. A “high-Ca diet” was based on the maintenance chow Ssniff R/M-H with additional 1.00% Ca^2+^ and 1.25% phosphor (P) (additional 0.55% P), using CaCO_3_ and Ca(H_2_PO_4_)_2_ to prevent hypercalcemia-induced phosphor deficiency (7). Mice given a “high-Zn diet” were maintained on the standard maintenance chow Ssniff R/M-H and drinking water containing 0.10% Zn^2+^ (ZnSO_4_). A combined “high-Mg/Ca/Zn diet” was generated using the standard Ssniff R/M-H chow with added 0.53% Mg^2+^, 1.00% Ca^2+^, and drinking water containing 0.10% Zn^2+^. The supplemented mice were inspected twice per day. Dead individuals and animals which were expected to die within next 24 h and killed for ethical reasons were genotyped as described above. Kaplan–Meier distributions and statistical analysis of the survival data of the mice maintained on the regular diet vs. individuals from treated groups were computed by the log-rank test using GraphPad Prism 7.3.

## Supplementary Material

Supplementary File

Supplementary File

Supplementary File
