# Supplementary material for: TRPM7 is the central gatekeeper of intestinal mineral absorption essential for postnatal survival
Source: Proc Natl Acad Sci U S A. 2019 Feb 15;116(10):4706–15. doi: 10.1073/pnas.1810633116 (PMC6410795; doi:10.1073/pnas.1810633116)

PNAS
www.pnas.org

Supplementary Information for

TRPM7 is the central gatekeeper of intestinal mineral absorption essential for postnatal survival

Lorenz Mittermeier, Lusine Demirkhanyan, Benjamin Stadlbauer, Andreas Breit, Camilla Recordati, Anne Hilgendorff, Masayuki Matsushita, Attila Braun, David G. Simmons, Eleonora Zakharian, Thomas Gudermann and Vladimir Chubanov

Corresponding authors: Thomas Gudermann and Vladimir Chubanov

Email: vladimir.chubanov@lrz.uni-muenchen.de; thomas.gudermann@lrz.uni-muenchen.de

This PDF file includes:

Supplementary text

Captions for databases S1 to S2

Tables S1 to S3

References for SI reference citations

Figures S1 to S8

www.pnas.org/cgi/doi/10.1073/pnas.1810633116

Supplementary Information Text

Supplementary Materials and Methods

Determination of cellular content of main elements and $^{65}Zn^{2+}$ uptake in HAP1 cells
WT and *TRPM7*-deficient human haploid leukaemia (HAP1) cells were described previously (1). The cells were cultured in Iscove's Modified Dulbecco's Medium (IMDM) supplemented with 10% FBS, 100 µg/ml streptomycin, 100 U/ml penicillin and 10 mM $MgCl_2$ (all from Thermo Fisher Scientific). Cells were maintained in a humidified cell culture incubator (Heraeus, Thermo Fisher Scientific) at 37°C and 5% CO_2.

Cellular content of main elements in dry pellets of HAP1 cells were determined by inductively coupled plasma mass spectrometry (ICP-MS) in ALS Scandinavia (Sweden) as reported previously (1-4) with a few modifications. HAP1 cells of each genotype were grown in T175 cm^2 flasks (Sarstedt) in the IMDM-based medium as described above. At ~50% confluence the medium was replaced with fresh medium without 10 mM $MgCl_2$ and the cells were cultured for additional 24h. Next, cells were washed with PBS, disaggregated by trypsinization and collected in 50 ml plastic tubes (Sarstedt). After centrifugation (3 min, 1000 rpm), the cell pellet was resuspended in 1 ml PBS and passed to a fresh 1.5 ml tube. The cell suspension was centrifuged (3 min, 3500 rpm), supernatant was completely removed and the cell pellet was dried overnight at 70°C. The dried cell pellet was analysed by ICP-MS in ALS Scandinavia (Sweden). The experiment was repeated 4 times. Data are presented as means ± standard error of the mean (SEM). Data were compared by an unpaired Student's t-test. Significance was accepted at $P \leq 0.05$.

To examine $^{65}Zn^{2+}$ uptake, HAP1 cells of each genotype were grown in T175 cm^2 flasks (Sarstedt) in the IMDM-based medium as described above. At ~90% confluence, the cells were washed 1x with PBS at room temperature (RT) and disaggregated by a cell scraper in the presence of RT PBS. After centrifugation (3 min, 1200 g), the cell pellet was resuspended in RT Hepes buffered saline (HBS) containing 150 mM NaCl, 5.4 mM KCl, 5 mM Hepes, 10 mM Glucose, 2 mM $CaCl_2$, 1 mM $MgCl_2$; pH=7.4 at a density of $5x10^6$ cells/ml. Aliquots of the cell suspensions were used for determination of protein content using Roti-Quant reagent (Carl Roth). HAP1 cells of each genotype were seeded in 2 ml-riplates (grade SW PP; Ritter) at a density of $2x10^5$ cells/well in 900 µl HBS containing 2 µM ^{65}Zn (Perkin Elmer) and incubated at 37°C for different time intervals as outlined in Fig. 1B. Incubation was terminated by harvesting of cells on 1.0 µm filters (grade GF/B, Whatman) using a Brandel cell harvester 9600-X (Brandel). The filters were quickly washed 4x with 2 ml deionized water. Afterwards, the filters were placed in 20 ml super polyethylene vials (Perkin Elmer) containing 5 ml Rotiszint solution (Carl Roth), and $^{65}Zn^{2+}$ radioactivity was measured using Wallac 1414 Win spectral liquid scintillation counter (Perkin Elmer). Blank values were determined by the omission of cells from the procedure described above. The experiment was repeated 6 times. The obtained datasets were fitted using nonlinear (least-squares) regression analysis (GraphPad Prism 6.0 software) and one-phase exponential association equation:

$$B = Bmax(1 - e^{-Kt})$$

with *B* being cellular content of $^{65}Zn^{2+}$ (dpm/µg protein), *t* - time (min), B_{max} - a maximum $^{65}Zn^{2+}$ level at equilibrium (dpm/µg protein); *K* - a rate constant (min^{-1}). Statistical assessment was performed using the extra sum-of-squares F test (GraphPad Prism 7.3) with the threshold $P \leq 0.05$.

Mouse strains and genotyping procedures

Mice strains were genotyped using PCR analysis of DNA isolated from tail fragments. DNA was extracted using the Mouse Direct PCR Kit (Biotool). DNA samples were analysed by PCR using a set of allele-specific oligonucleotides (Metabion) and M-PCR OPTI^{TM} kit (Biotool).

Trpm7^{fl} allele (Table S1) was analyzed using primers *Trpm7^{fl}*-Forward 5'-CACTTATGAACTCAAAAACTGGA-3' and *Trpm7^{fl}*-Reverse 5'-CGGAATTCGATATCGCACTAAC-3' with PCR settings: 94°C 5', 94°C 30'', 52°C 30'', 72°C 1', 35 cycles, 72°C 5'. *Trpm7^{WT}* allele was analyzed using primers *Trpm7^{+}*-Forward 5'-CACTTATGAACTCAAAAACTGGA-3' and *Trpm7^{+}*-Reverse 5'-GGATACATTCACTCAGCACTA -3' with PCR settings: 94°C 5', 94°C 30'', 57°C 30'', 72°C 1', 35 cycles, 72°C 5'. Amplified PCR products (529 bp for *Trpm7^{fl}*, 530 for *Trpm7^{WT}*) were visualised by agarose gel electrophoresis.

Inheritance of *Villin1-Cre* transgene (Table S1) was determined by PCR using *Villin1-Cre* Forward 5'-CATGTCCATCAGGTTCTTGC-3' and *Villin1-Cre* Reverse 5'-TTCTCCTCTAGGCTCGTCCA-3' with PCR settings: 94°C 2', 94°C 15'', 50°C 15'', 72°C 10'', 30 cycles, 72°C 2'. *Ksp1.3-Cre* allele (Table S1) was analyzed using primers *Ksp1.3-Cre*-Forward 5'-GCAGATCTGGCTCTCCAAAG-3' and *Ksp1.3-Cre* Reverse 5'-AGGCAAATTTTGGTGTACGG-3' with PCR settings: 94°C 2', 94°C 20'', 60°C 15'', 72°C 20'', 30 cycles, 72°C 2'. Amplified PCR products (195 bp for *Villin1-Cre*, and 420 bp for *Ksp1.3-Cre*) were visualised by agarose gel electrophoresis.

Trpm7^{R} allele (Table S1) was analyzed using primers *Trpm7^{R}*-Forward 5'-AATGGGAGGTGGTTTACG-3' and *Trpm7^{R}*-Reverse 5'-CTCAGACAGCTTACAGTC-3' with PCR settings: 94°C 5', 94°C 30'', 66°C 30'', 72°C 1', 40 cycles, 72°C 5'. The PCR product was digested for 16 h at 65°C with TruI restriction enzyme (Thermo Scientific) and were visualised by agarose gel electrophoresis. PCR amplicon from *Trpm7^{+}* allele was cleaved by TruI into 120 and 85 bp fragments, whereas PCR amplicon from *Trpm7^{R}* allele (205 bp) remained undigested.

Collection of specimens and determination of main elements

Collection of serum and bones (right tibias) was performed as described previously (1). To determine urinary excretion rates of main minerals, mice were maintained for 24h in individual metabolic cages (Acme Metal Products, USA) under housing conditions as described above and supplied *ad libitum* with drinking water and chow. After 24h, the urine produced was collected and stored at -80°C. Content of main elements was determined by inductively coupled plasma mass spectrometry (ICP-MS) by ALS Scandinavia (Sweden) as reported earlier (1-4).

Determination of parathyroid hormone (PTH 1-84), 1,25-dihydroxyvitamin D, thyroxine (T4), insulin-like growth factor 1 (IGF1) and interleukin 6 (IL-6)

Serum samples were collected from P5 *Trpm7$^{fl/fl}$* and *Trpm7$^{fl/fl}$;Villin1-Cre* littermates. Concentrations of PTH 1-84 were measured by a Mouse PTH 1-84 ELISA Kit (Immutopics). Samples with PTH contents below the assay sensitivity of 0.425 pM were defined to this level. Concentrations of 1,25-dihydroxyvitamin D were determined using a 1,25-Dihydroxy Vitamin D EIA (Immunodiagnostic Systems). To obtain the sufficient amount of serum, samples were pooled from 3-7 animals. Levels of T4 were determined by a Thyroxine (Mouse/Rat) ELISA Kit (BioVision). For analysis of IGF1 we used the Mouse/Rat IGF-1 Quantikine ELISA Kit (R&D

Systems). For determination of IL-6 we used Q-Plex^{TM} Mouse multiplexed ELISA (Quansys Biosciences).

Isolation of RNA and qPCR analysis

Villi were isolated according to (5) with a few modifications. Whole intestines were dissected from P5 *Trpm7$^{fl/fl}$* and *Trpm7$^{fl/fl}$;Villin1-Cre* mice, cut longitudinally and flushed 1x with ice-cold PBS and 3x with ice-cold PBS containing 1 mM Dithiothreitol (DTT). Next, the individual tissues were incubated for 15 min in 4 ml ice-cold solution containing 96 mM NaCl, 27 mM Na-citrate, 1.5 mM KCl, 8 mM KH_2PO_4, 5.6 mM Na_2HPO_4 (pH 7.4). Afterword, the tissues were transferred to 1.5 ml PBS containing 1.5 mM ethylenediaminetetraacetic acid (EDTA), 0.5 mM DTT, 1 mg/ml bovine serum albumin (BSA) and incubated for 15 min at 10 °C at a constant rotation (250 rpm). The tissues were vortexed thoroughly 5 times for 10 s. The tissues were removed and released villi were collected by centrifugation (1 min, 13.000 g, 4 °C). The obtained villi pellets were flash frozen in liquid nitrogen and stored at -80 °C. Aliquots of villi samples were examined using an Olympus CX41 microscope and Cell Imaging software (Olympus, Germany). Villi RNA was extracted with GenElute^{TM} Mammalian Total RNA Miniprep Kit (Sigma-Aldrich). The used PCR primer pairs (Metabion) are outlined in Table S3. First strand cDNA synthesis was generated using RevertAid Reverse Transcription Kit (Thermo Fisher). qPCR reactions were performed using the Thermo Scientific ABsolute qPCR SYBR Green Mix (Thermo Scientific) and a LightCycler 480 (Roche) with the following PCR settings: 95°C 15', 95°C 15', 60°C 15'', 72°C 30''. The cycle thresholds (Ct) of test and reference genes (*Hprt* or *Ywhaz)* were calculated using LightCyler 480 software (Roche). The relative mRNA expression levels were calculated using the $2^{-\Delta\Delta CT}$ approach. Statistical analysis was performed using an unpaired Student's t-test.

Histological examination of tissues

Intestinal segments were obtained from P1 *Trpm7$^{fl/fl}$* (n=3) and *Trpm7$^{fl/fl}$;Villin1-Cre* (n=3) littermates. Spleens and thymuses were examined in P5 *Trpm7$^{fl/fl}$* (n=5) and *Trpm7$^{fl/fl}$;Villin1-Cre* (n=5) littermates. Tissues were fixed at 4°C in PBS containing 4% paraformaldehyde (PFA), routinely processed for paraffin embedding, sectioned at 4 µm thickness, stained with hematoxylin and eosin (H&E, Mayer's haematoxylin, cat. No. C0302; Eosin G, cat. No. C0362, Diapath, Martinengo, Bergamo, Italy) and evaluated under a light microscope (Leica, DM2500)

In situ hybridization (ISH)

Kidneys were obtained from 8-week-old *Trpm7$^{fl/fl}$* (n=3) and *Trpm7$^{fl/fl}$;Ksp-Cre* (n=3) littermates. All procedures up to the hybridization step were performed with Diethyldicarbonat (DEPC)-treated solutions. The tissues were incubated in PBS containing 4% Paraformaldehyde (PFA) for 24h at 4°C and afterwards were stored in PBS with 0.4% PFA until embedding. Tissues were dehydrated through an ethanol series, cleared in xylene and embedded in paraffin. 7 µm sections were mounted on SuperFrost Plus slides and dried O/N at 37°C. cDNA templates for the production of cRNA *Trpm7* probes were produced by PCR with the following primers: Forward: 5'-aattaaccctcactaaagggTGGCAGTTGAATTACTGGAACA-3'; Reverse: 5'-taatacgactcactatagggTCATATTCAGCCGTCCCATC-3'. Primers contained T7 (reverse primer lower case) or T3 (forward primer lower case) RNA polymerase binding sites. PCR products were gel purified (Qiagen Gel Extraction Kit) and sequence verified (ARGF). Digoxigenin (DIG) labelled cRNA probes were synthesized according the manufacturer's instructions (Roche, 10x

DIG RNA labelling kit). Preparation of tissue sections and ISH procedures were performed as previously described (6). Slides were imaged by an Aperio slide scanner and analysed using ImageScope software.

Immunohistochemistry

Duodenal samples were dissected from eight 8-week-old and P5 mice and fixed in 4% (w/v) PFA (Electron Microscopy Sciences) in PBS (pH 7.4) for 2h at room temperature (RT), followed by incubation in PBS for 1h at RT. Next, tissues were incubated in 18% sucrose (Sigma-Aldrich) in PBS for 24h at 4°C and embedded in Jung tissue freezing medium (Leica Microsystems, Germany). 16 µm cryosections were produced by a CM 3050S cryotom (Leica Microsystems, Germany), mounted on Superfrost Plus slides (Menzel-Gläser) and air-dried for 20 min. After washing in PBS (3x 5 min, RT), sections were incubated in PBS containing 10% goat serum, 3% BSA and 0.05% Tween-20 (PBST) for 1h at RT. The rabbit anti-TRPM7 antibody (AB15562 Millipore; 1.7 µg/ml in PBST) was applied overnight at 4°C. Afterwards sections were washed in PBST (3x 5 min, RT) and a goat anti-rabbit antibody conjugated with Alexa Fluor 488 (Life Technologies; 1 µg/ml in PBST) was applied for 2h at RT. After washing in PBS (3x 5 min, RT), sections were stained with Hoechst 33342 (Life Technologies; 10 µg/ml in PBST) and mounted under Dako Fluorescent Mounting Medium (Agilent Dako). Differential interference contrast (DIC) and confocal images were obtained with a confocal laser scanning microscope LSM 880 AxioObserver (Carl Zeiss). We used a C-Apochromat 63x/1.2 W objective, 405 nm and 488 nm excitation wavelengths, and 410–501 nm and 493–630 nm filters. Acquired DIC and confocal images were analysed using the ZEN2.3 software (Carl Zeiss).

Whole-genome profiling of the villi transcriptome

Small intestines were obtained from P5 *Trpm7*$^{fl/fl}$ (n=3) and *Trpm7*$^{fl/fl}$*;Villin1-Cre* (n=3) littermates, cut along the lengthwise axis, washed 1x with ice-cold PBS and placed in RNA*later* RNA reagent (QIAGEN). Villi were mechanically disassociated by vortexing of the tissues (20 times, 10s at 3000 rpm, RT). The tissues were removed and released villi were collected by centrifugation (1 min, 17.000 g, RT). RNA was extracted using GenElute Mammalian Total RNA Miniprep kit (Sigma-Aldrich). Whole genome profiling was performed using a GeneChip Mouse Gene 1.0 ST Array (Affymetrix) at Source Bioscience (Berlin, Germany) as described previously (1). Processing of the array data, including quality assessment, background correction and normalization were performed with the Affymetrix Expression Console (version 1.4.0). Differential expression analysis was performed with DNASTAR ArrayStrar 11.0 software (Supporting file 1). P-values (Student's t-test) were adjusted for multiple testing with the Benjamini-Hochberg method for controlling the false discovery rate (FDR). Analysis of the affected gene networks was performed by the Ingenuity pathway analysis (IPA) computer environment using a set of 455 transcripts with ≥2.0 fold change at P≤0.05 (Supporting file 2). To compare the Ingenuity Canonical Pathways highly affected in villi of *Trpm7*$^{fl/fl}$*;Villin1-Cre* versus those in livers of *Trpm6* null mice (1), the IPA Comparison Analysis option was used as outlined in Fig. 3I. Microarray data were deposited in NCBI Gene Expression Omnibus (GEO) (GSE110613).

Supplementary Figures and Figure Legends

Figure S1. Elementary contents of Ca, Mg and Zn in human haploid leukaemia (HAP1) cells deficient in *TRPM7*.
(A, B) Total elementary contents of divalent metals shown in Fig. 1A were normalized to total elementary contents of rubidium (Rb) (A) or phosphor (P) (B). Results are represented as mean±SEM. *** - P≤0.001; ** - P≤0.01; n.s. – not significantly different (Student's t-test). n – number of independent measurements. **(C)** Determination of elementary levels of Zn in WT and KO HAP1 cells. Dried cell pellets were obtained from WT and KO HAP1 cells cultured in standard cell culture medium or in a medium containing an additional 50 µM $ZnSO_4$ for 24h and analysed by ICP-MS. Total elementary Zn contents were normalized to dry pellet weight and represented as mean±SEM of n=4 independent measurements. ** - P≤0.01; * - P≤0.05; n.s. – not significantly different (One-Way ANOVA).

Figure S2. Effects of naltriben and NS8593 on TRPM7 channel activity in lipid bilayers.
(A) Representative traces obtained for TRPM7 inhibition by 1 µM NS8593 after channel activation by 2.5 µM naltriben in the presence of 2.5 µM PIP_2 at +150 mV (N=4 independent experiments with n=9,793 evens). The closed and open states of TRPM7 are outlined by (*c*) and (*o*), respectively. **(B)** Stimulatory effect of 2.5 µM naltriben on P_o (mean±SEM) of TRPM7 in the presence of 2.5 µM PIP_2 (N=9, n=34,355). **(C)** Inhibitory effect of 1 µM NS8593 on P_o (mean±SEM) of TRPM7 measured at +150 mV in the presence of 2.5 µM PIP_2 and 2.5 µM naltriben (N=3, n=13,989). Experiments were performed in symmetric ionic conditions (see Methods). *** - P≤0.001; ** - P≤0.01 (One-Way ANOVA).

Figure S3. Analysis of mice with kidney-restricted inactivation of *Trpm7*.
(A) ISH in paraffin sections obtained from 8-week-old *Trpm7$^{fl/fl}$* (Control) and *Trpm7$^{fl/fl}$;Ksp1.3-Cre* (Kidney KO) littermate males using an antisense probe for *Trpm7.* Arrows indicate the tubule segments positive for WT *Trpm7* transcript and morphologically resembling DCT. Representative images are shown (n=2 tissues per genotype). **(B)** ICP-MS analysis of elementary levels (mean±SEM) of Ca *(left panel)*, Mg *(middle panel)* and Zn *(right panel)* in serum of 8-week-old male littermates. **(C)** 24h urinary excretion rate of Ca^{2+} *(left panel)*, Mg^{2+} *(middle panel)* and Zn^{2+} *(right panel)* of 8-week-old male littermates. **(D)** Relative expression levels of *Trpm6*, *Claudin-16*, *ZnT1*, *ZnT2* and *Trpv5* were studied in mRNA extracts from whole kidneys of 8-week-old littermate males examined by qPCR and *Ywhaz* as a reference transcript. Results are shown as fold-change (mean±SEM) in *Trpm7$^{fl/fl}$;Ksp1.3-Cre versus Trpm7$^{fl/fl}$* mice. n.s. – not significantly different (Student's t-test); n – number of mice examined.

Figure S4. Histology of duodenum, jejunum, ileum and colon in mice with intestine-restricted inactivation of *Trpm7* at P1.
Hematoxylin-eosin staining of paraffin embedded tissue sections of P1 *Trpm7$^{fl/fl}$* (Control) and *Trpm7$^{fl/fl}$;Villin1-Cre* (Intestine KO) littermates. Representative images are shown (n=3 tissues per genotype). The triangle indicates duodenal erosion. Arrows point at vacuolization of enterocytes in jejunum.

Figure S5. Immunostaining of duodenum cryosections using a TRPM7-specific antibody.
(A) Confocal scans of TRPM7 immunoreactivity (Alexa488) and Hoechst staining (Hoechst), and differential interference contrast (DIC) images acquired from tissue sections of 8-week-old or P5 *Trpm7*$^{fl/fl}$ (Control) mice and P5 *Trpm7*$^{fl/fl}$*;Villin1-Cre* (Intestine KO) mice. Representative images are shown (n=2 tissues per genotype). Triangles indicate labelling of the apical surface of enterocytes observed only in control tissues.

Figure S6. Analysis of elementary levels of sodium (Na) and potassium (K) in serum and bones of P5 mice with intestine-restricted inactivation of *Trpm7*.
ICP-MS analysis of elementary levels (mean±SEM) of Na *(left panel)* and K *(right panel)* in the serum **(A)** and bones **(B)** of *Trpm7*$^{fl/fl}$ (Control) and *Trpm7*$^{fl/fl}$*;Villin1-Cre* (Intestine KO) littermates. In (B) total elementary contents were normalized to dry bone weights. ** - P≤0.01; n.s. – not significantly different (Student's t-test); n – number of mice examined per data point.

Figure S7. Morphological examination of thymus and spleen in mice with intestine-restricted inactivation of *Trpm7* at P5.
(A, D) Overall appearance, **(B, E)** weight of tissues normalized to body size and **(C, F)** hematoxylin-eosin staining of paraffin embedded sections of thymus (A-C) and spleen (D-F) from P5 *Trpm7*$^{fl/fl}$ (Control) and *Trpm7*$^{fl/fl}$*;Villin1-Cre* (Intestine KO) littermates. The triangle indicates the medulla region detectable only in WT thymus and points at the white pulp, which can only be observed in WT spleens. Representative images are shown (n=5 tissues per genotype). ** - P≤0.01 (Student's t-test).

Figure S8. Assessment of organismal balance of divalent cations in mice with a global 'kinase-dead' mutation in *Trpm7*.
(A, B) ICP-MS analysis of elementary levels (mean±SEM) of Ca *(left panel)*, Mg *(middle panel)* and Zn *(right panel)* in serum (A) and bones (B) of 8-week-old *Trpm7*$^{WT/WT}$ (+/+) and *Trpm7*$^{R/R}$ (*R/R*) littermate males. In (B) total elementary contents were normalized to dry bone weights. Results are represented as mean±SEM. * - P≤0.05; n.s. – not significantly different (Student's t-test); n – number of mice examined.

Captions for Supplementary Datasets

Supplementary datasets 1. Whole-genome profiling of transcripts altered in *Trpm7*-deficient villi. Excel file contains 1 worksheet: Excel file shows genome-wide assessment of transcriptomes in villi isolated from control (n=3) and *Trpm7*-deficient (n=3) mice.

Supplementary datasets 2.
Ingenuity Pathway Analysis (IPA) analysis of transcriptome altered in villi of *Trpm7*-deficient mice.
Excel file contains 2 worksheets:
(1) 455 up- and down-regulated transcripts in *Trpm7*-deficient (n=3) versus control (n=3) mice with ≥2.0-fold change and the false discovery rate (FDR) P≤0.05;
(2) IPA Canonical Pathways using 455 up- and down-regulated transcripts outlined in (1).

Supplementary tables

Table S1. Generation of the mice with global and tissue-restricted mutations of *Trpm7.*

Targeted tissue	Breeding strategy	Expected F1 outcome[a]
Intestine	♂*Trpm7*$^{fl/WT}$*;Villin1-Cre* x ♀*Trpm7*$^{fl/fl}$	<u>25% *Trpm7*$^{fl/fl}$*;Villin1-Cre*</u>[b]<br>25% *Trpm7*$^{fl/fl}$<br>25% *Trpm7*$^{fl/WT}$*;Villin1-Cre*<br>25% *Trpm7*$^{fl/WT}$
Kidney	♂*Trpm7*$^{fl/WT}$*;Ksp1.3-Cre* x ♀*Trpm7*$^{fl/fl}$	<u>25% *Trpm7*$^{fl/fl}$*;Ksp1.3-Cre*</u>[b]<br>25% *Trpm7*$^{fl/fl}$<br>25% *Trpm7*$^{fl/WT}$*;Ksp1.3-Cre*<br>25% *Trpm7*$^{fl/WT}$
Global	♂*Trpm7*$^{R/WT}$ x ♀*Trpm7* $^{R/WT}$	<u>25% *Trpm7*$^{R/R}$</u><br>50% *Trpm7*$^{R/WT}$<br>25% *Trpm7*$^{WT/WT}$

[a]Genotypes were determined using genomic DNA extracted from tail fragments.
[b]Individuals were assumed to be homozygous for *Trpm7*$^{\Delta 17}$ allele in the cells expressing Cre.

Table S2. Dietary regimes used for survival experiments.

Diet	Chow	Drinking water
Control	Ssniff M-Z*	Deionized water
High Mg	Ssniff M-Z + 0.53% Mg^{2+}	Deionized water
High Ca	Ssniff M-Z + 1.00% Ca^{2+}	Deionized water
High Zn	Ssniff M-Z	Deionized water + 0.1% Zn^{2+}
High Mg/Ca/Zn	Ssniff M-Z + 0.53% Mg^{2+} + 1.00% Ca^{2+}	Deionized water + 0.1% Zn^{2+}

*Ssniff M-Z contained 0.22% Mg^{2+}, 1.00% Ca^{2+} and 0.089% Zn^{2+}.

Table S3. Primer used in the present study.

Primer	Sequence	Amplicon
Abcc1 for	5`-GGA CAA GGT GGA GGG ACA T-3`	84 bp
Abcc1 rev	5`-CGG AGA GAG TCA TTC TGA ATC C-3`	
Abcc2 for	5`-TTT CCT GGA TTA CCT CCA ACC-3`	76 bp
Abcc2 rev	5`-GCC GAG CAG AAG ACA ATC A-3`	
Alpi for	5`-CGT AAT TGG TAC TCA GAT GCA GA-3`	113 bp
Alpi rev	5`-CCT CCA CCG AGG ATC ACA-3`	
Calbindin-D_{9k} for	5`-CCT GCA GAA ATG AAG AGC ATT TT-3`	175 bp
Calbindin-D_{9k} rev	5`-CTC CAT CGC CAT TCT TAT CCA-3`	
Claudin-16 for	5`-GGT TGC TTT TTG GCA GGA-3`	74 bp
Claudin-16 rev	5`-TAG TTC CTC TCA GGC CCA AC-3`	
Cyp27b1 for	5`-AGT GGG GAA TGT GAC AGA GC-3`	61 bp
Cyp27b1 rev	5`-GGA GAG CGT ATT GGA TAC CG-3`	
Dclk1 for	5`-TCT GTG GCA CCC CAA CAT A-3`	66 bp
Dclk1 rev	5`-CCA CCT TGA GGC CAT ATC C-3`;	
Fbkp5 for	5`-AGG GCA CCA GTA ACA ATG GA-3`	110 bp
Fbkp5 rev	5`-CCC CAC TCT TTT GAC AAT CTT T-3`;	
Fos for	5`-CAG CCT TTC CTA CTA CCA TTC C-3`	86 bp
Fos rev	5`-ACA GAT CTG CGC AAA AGT CC-3`	
Gclc for	5`-AGA TGA TAG AAC ACG GGA GGA G-3`	62 bp
Gclc rev	5`-TGA TCC TAA AGC GAT TGT TCT TC-3`	
Gpx2 for	5`-GTT CTC GGC TTC CCT TGC-3`	64 bp
Gpx2 rev	5`-TCA GGA TCT CCT CGT TCT GAC-3`	
Gsr for	5`-ACT ATG ACA ACA TCC CTA CTG TGG-3`	89 bp
Gsr rev	5`-CCC ATA CTT ATG AAC AGC TTC GT-3`	
Gss for	5`-TGT CCA ATA ACC CCA GCA AG-3`	87 bp
Gss rev	5`-TCA GTA GCA CCA CCG CAT T-3`	
Gsta1 for	5`-CTT CTG ACC CCT TTC CCT CT-3`	87 bp
Gsta1 rev	5`-GCT GCC AGG CTG TAG GAA C-3`	
Gsta2 for	5`-TCT GAC CCC TTT CCC TCT G-3`	85 bp
Gsta2 rev	5`-GCT GCC AGG ATG TAG GAA CT-3`	

Table S3 continued

Gsta3 for	5`-TGG ACA ACT TCC CTC TCC TG-3`	107 bp
Gsta3 rev	5`-TGC GTC ATC AAA AGG CTT C-3`	
Gstm1 for	5`-GCA GCT CAT CAT GCT CTG TTA-3`	76 bp
Gstm1 rev	5`- TTT CTC AGG GAT GGT CTT CAA-3`	
Gstm3 for	5`-TTA TGG ACA CCC GCA TAC AG-3`	75 bp
Gstm3 rev	5`-TCA AGA ACT CTG GCT TCT GCT-3`	
Gstp1 for	5`-TGT CAC CCT CAT CTA CAC CAA C-3`	94 bp
Gstp1 rev	5`-GGA CAG CAG GGT CTC AAA AG-3`	
Hprt for	5`-CTC ATG GAC TGA TTA TGG ACA GG-3`	135 bp
Hprt rev	5`-TTA ATG TAA TCC AGC AGG TCA GC-3`	
Lgr5 for	5`-CTG CCC ATC ACA CTG TCA CT-3`	172 bp
Lgr5 rev	5`-GCA GAG GCG ATG TAG GAG AC-3`	
Lyz1 for	5`-GGC AAA ACC CCA AGA TCT AA-3`	104 bp
Lyz1 rev	5`-TCT CTC ACC ACC CTC TTT GC-3`	
Mgst2 for	5`-CGG ACG AGC AAG ACT AAA ACA-3`	113 bp
Mgst2 rev	5`-TGA ATA CAG GAT AAA ACT CCA AAG AG-3`	
Mki-67 for	5`-AGG GTA ACT CGT GGA ACC AA-3`	78 bp
Mki-67 rev	5`-TCT TAA CTT CTT GGT GCA TAC AAT G-3`	
Muc2 for	5`-GAC CTG ACA ATG TGC CCA GA-3`	66 bp
Muc2 rev	5`-GGC AAA CAC AGT CCT TGC AG-3`	
Nqo1 for	5`-AGC GTT CGG TAT TAC GAT CC-3`	68 bp
Nqo1 rev	5`-AGT ACA ATC AGG GCT CTT CTC G-3`	
Slc41a1 for	5`-CAT GGT GCT GGA CAT TGT G-3`	109 bp
Slc41a1 rev	5`-CAG GGT CAT TTC CAA GTT CC-3`	
Slc41a2 for	5`-GTT TAC ACG CCA GTT ATC AAC G-3`	77 bp
Slc41a2 rev	5`-TGG AGG TAG GTA GAA ATC CTG CT-3`	
Trpm6 for	5`-CAC CGC TTC CTC ACC ATC-3`	74 bp
Trpm6 rev	5`-CGC AAA AAT TTA TTA GTT GGT CCT-3`	
Trpm7 ex. 17 for	5`-AGTAATTCAACCTGCCTCAA-3`	287 bp
Trpm7 ex. 17 rev	5`-ATGGGTATCTCTTCTGTTATGTT-3`	

Table S3 continued

Trpv5 for	5`-ATT GAC GGA CCT GCC AAT TAC AGA G-3`	285 bp
Trpv5 rev	5`-GTG TTC AAC CCG TAA GAA CCA ACG-3`	
Trpv6 for	5`-ATC GAT GGC CCT GCG AAC T-3`	358 bp
Trpv6 rev	5`-CAG AGT AGA GGC CAT CTT GTT GCT G-3`	
Txndr1 for	5`-GAC TAA GCA GCA GCT GGA CA-3`	136 bp
Txndr1 rev	5`-CAT CCA CAC TGG GGC TTA AC-3`	
Vil1 for	5`-ATC TCC CTG AGG GTG TGG A-3`	62 bp
Vil1 rev	5`-AGT GAA GTC TTC GGT GGA CAG-3`	
Ywhaz for	5`-TAA AAG GTC TAA GGC CGC TTC-3`	60 bp
Ywhaz rev	5`-CAC CAC ACG CAC GAT GAC-3`	
Zip4 for	5`-ACT TTG TGG ACT TTG TGT TCA GG-3`	193 bp
Zip4 rev	5`-GAG TAT GGA GCT CAG AGT CTT GG-3`	
Zip5 for	5`-AGG ACC TAG TGA GCA ATC AGA GG-3`	155 bp
Zip5 rev	5`-TTC TCC AAG ATC CCT TTT GTT CC-3`	
ZnT1 for	5`-TGG ATG TAC AAG TAA ATG GGA ATC T-3`	62 bp
ZnT1 rev	5`-GTC TTC AGT ACA ACC CTT CCA GTT A-3`	
ZnT2 for	5`-CAG AAG GAT TCT GGA AGT CAC C-3`	211 bp
ZnT2 rev	5`-CGG GAA GAC ACC CAG AGG-3`	

Supplementary References

1. Chubanov V*, et al.* (2016) Epithelial magnesium transport by TRPM6 is essential for prenatal development and adult survival. *Elife* 5.
2. Rodushkin I & Odman F (2001) Application of inductively coupled plasma sector field mass spectrometry for elemental analysis of urine. *J Trace Elem Med Biol* 14(4):241-247.
3. Rodushkin I, Engstrom E, Stenberg A, & Baxter DC (2004) Determination of low-abundance elements at ultra-trace levels in urine and serum by inductively coupled plasma-sector field mass spectrometry. *Anal Bioanal Chem* 380(2):247-257.
4. Ryazanova LV*, et al.* (2014) Elucidating the role of the TRPM7 alpha-kinase: TRPM7 kinase inactivation leads to magnesium deprivation resistance phenotype in mice. *Sci Rep* 4:7599.
5. Kabiri Z*, et al.* (2014) Stroma provides an intestinal stem cell niche in the absence of epithelial Wnts. *Development* 141(11):2206-2215.
6. Dawson PA, Rakoczy J, & Simmons DG (2012) Placental, renal, and ileal sulfate transporter gene expression in mouse gestation. *Biol Reprod* 87(2):43.

Suppl. Figure 1

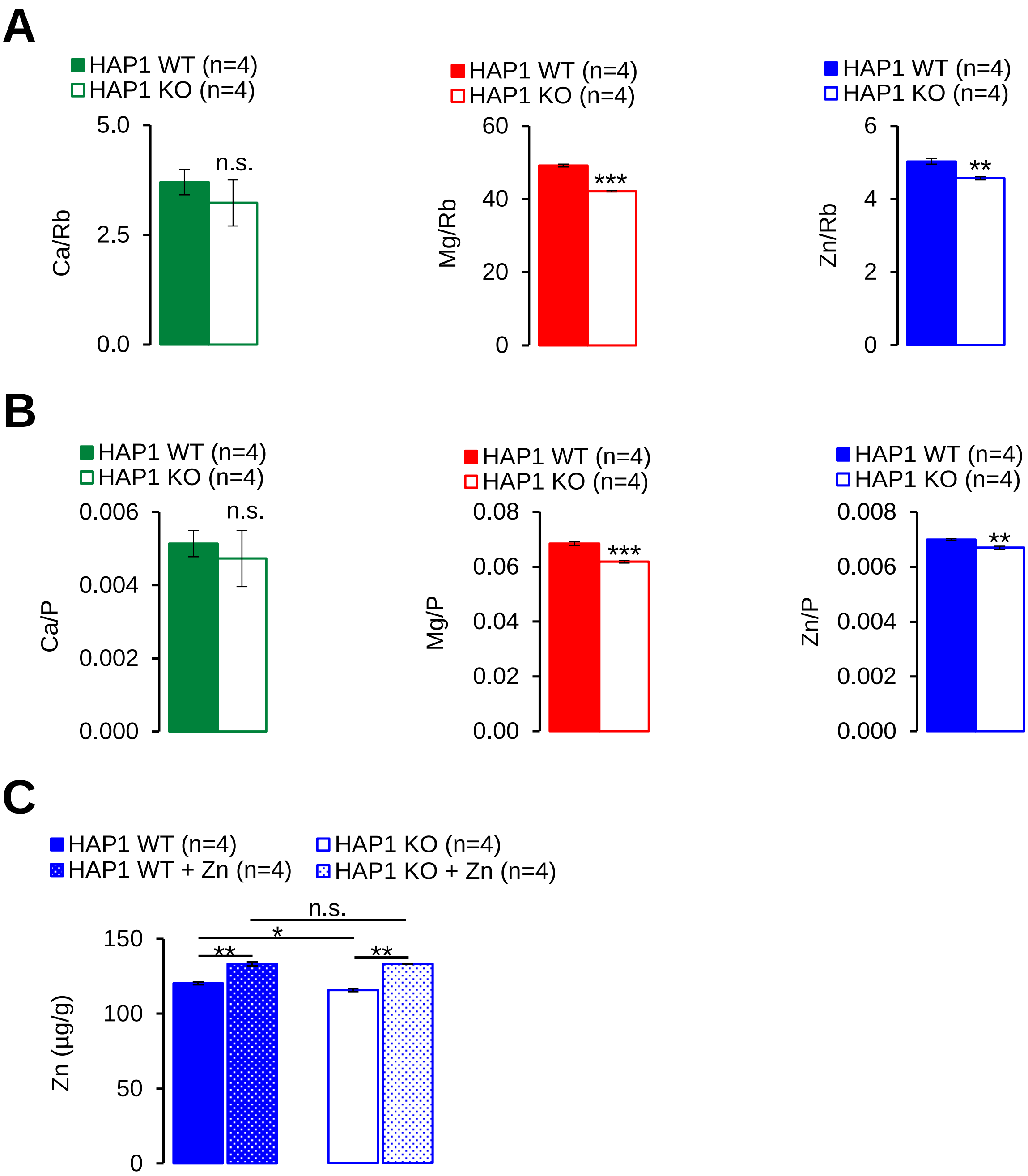

Suppl. Figure 2

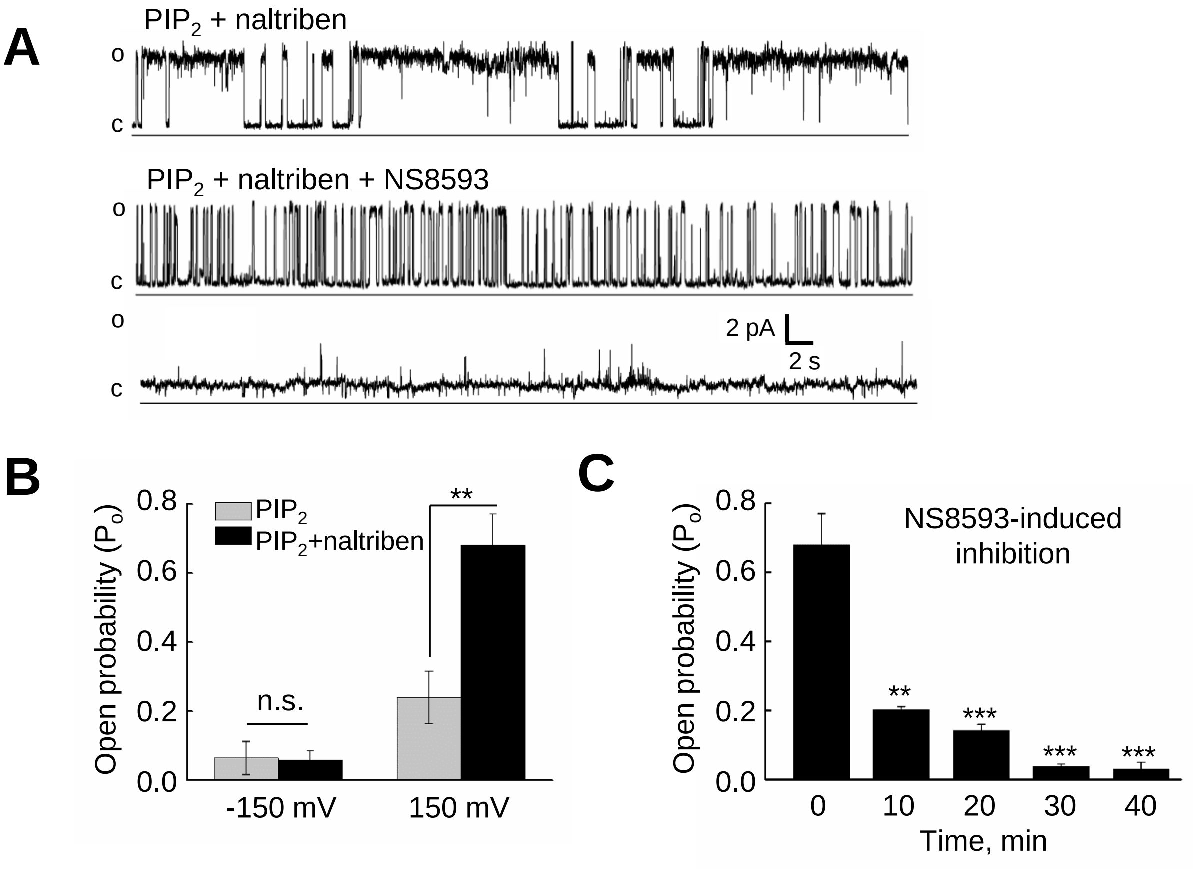

Suppl. Figure 3

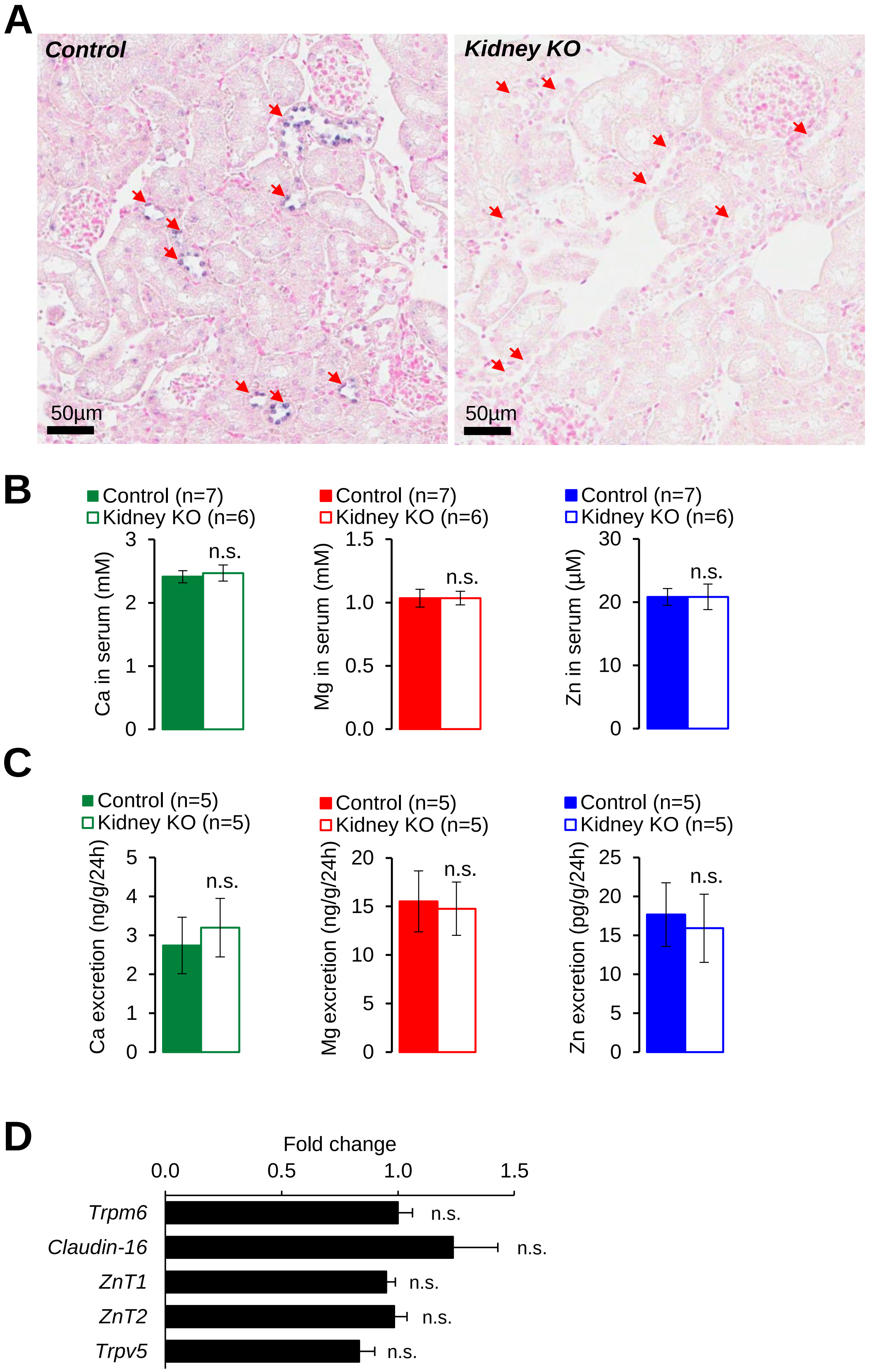

Suppl. Figure 4

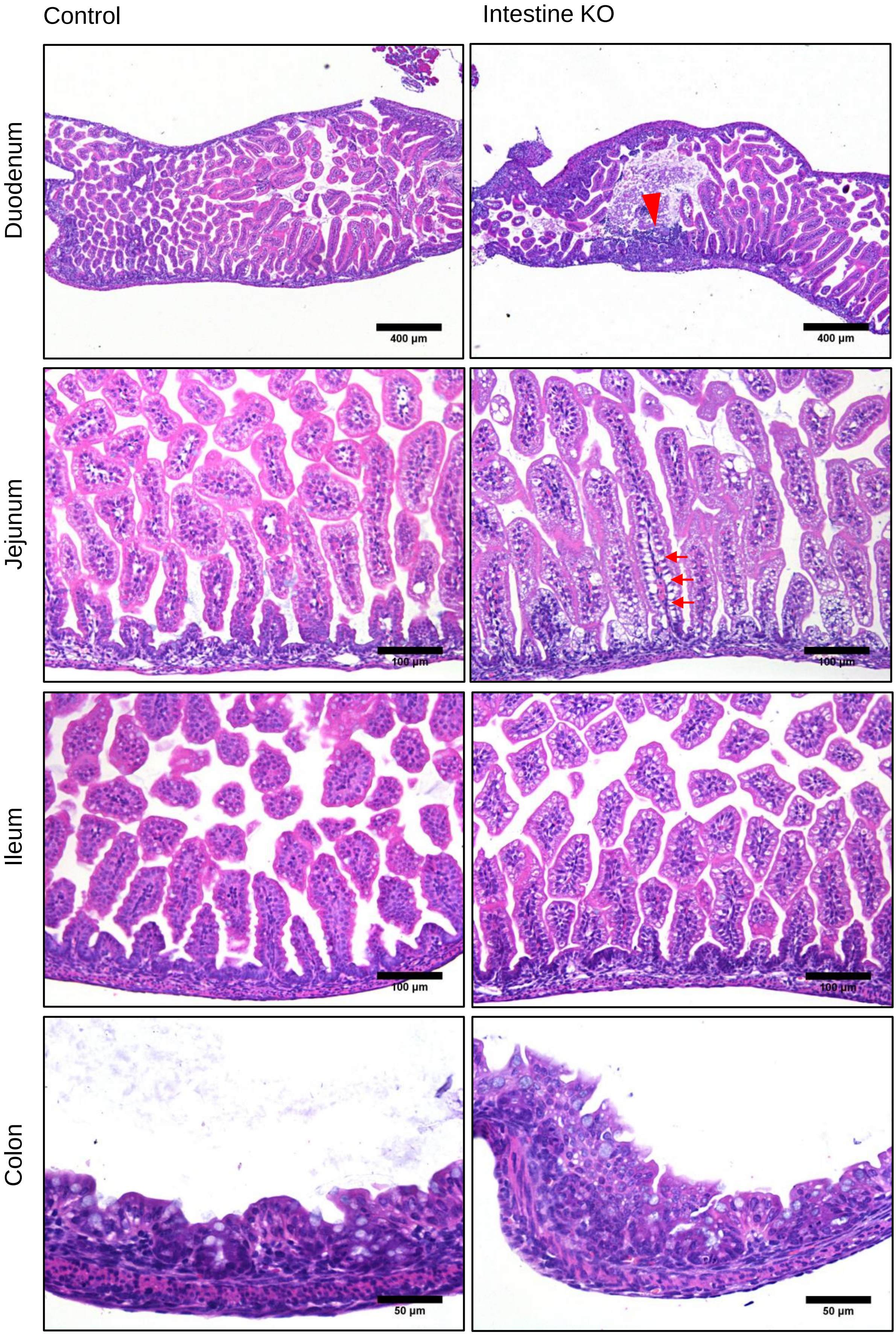

Suppl. Figure 5

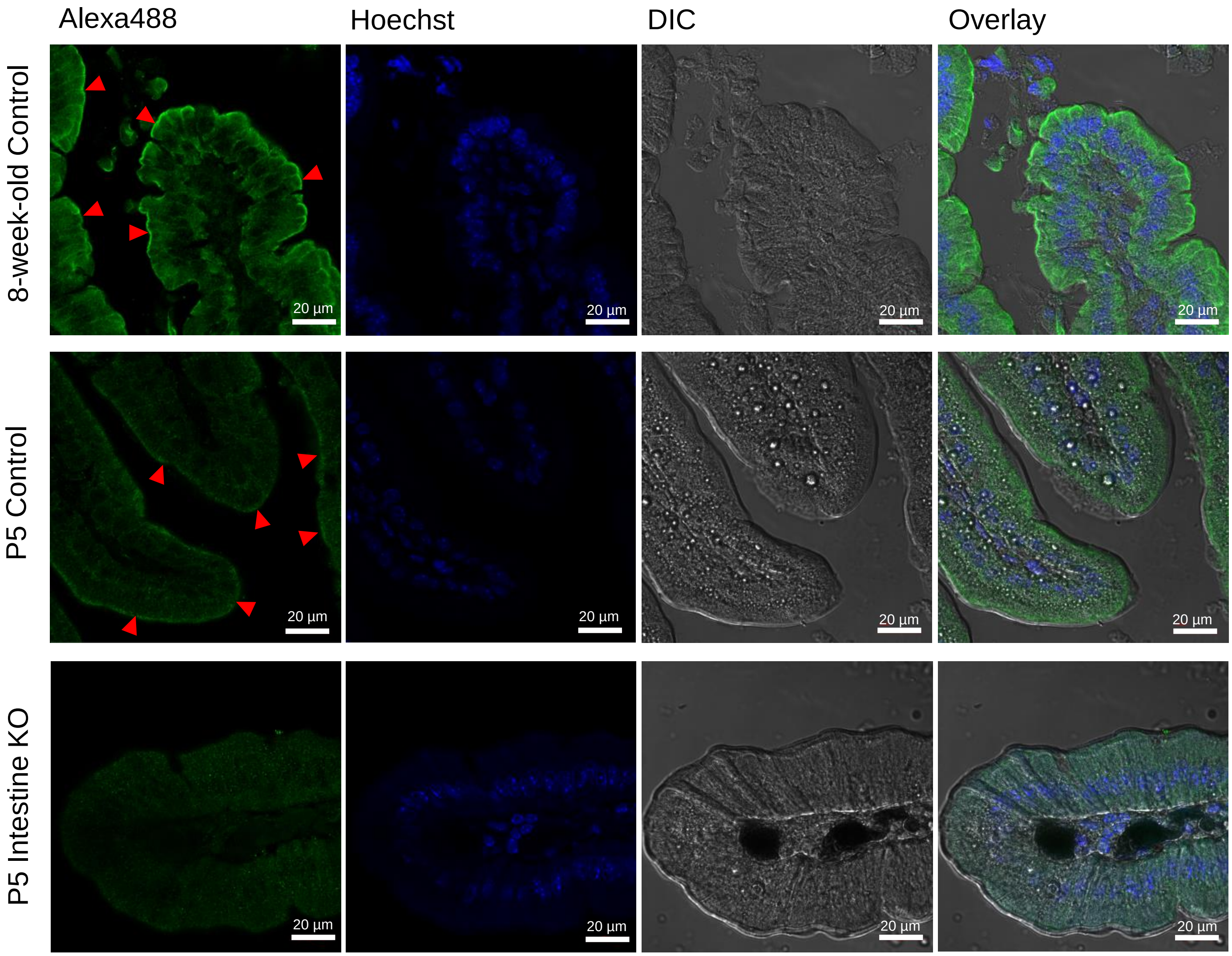

Suppl. Figure 6

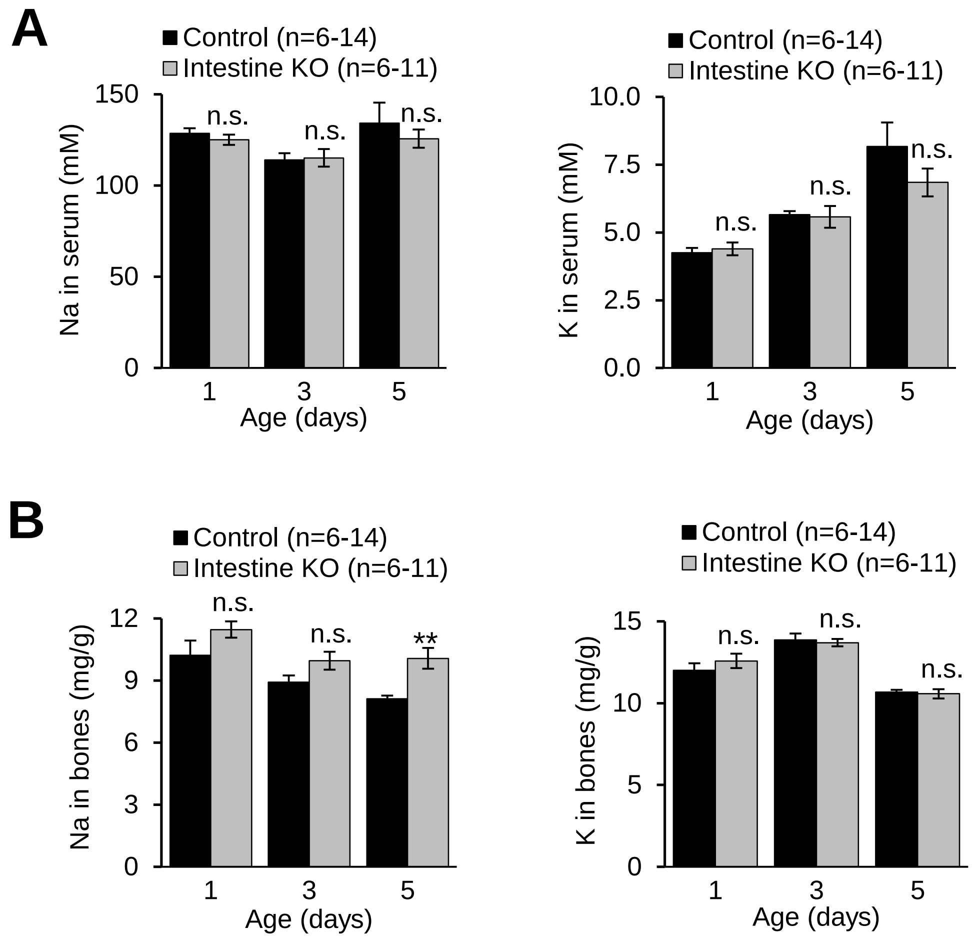

Suppl. Figure 7

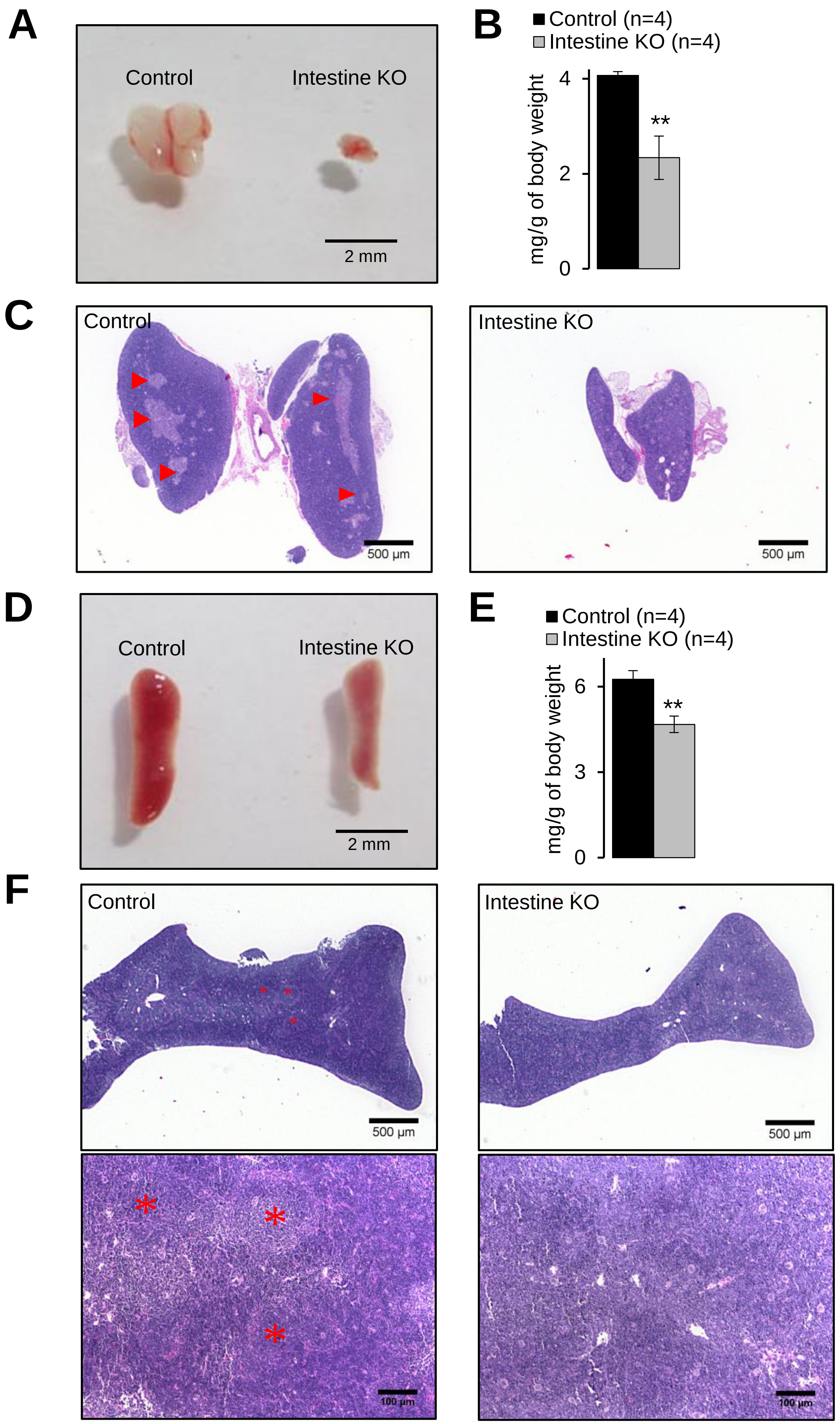

Suppl. Figure 8

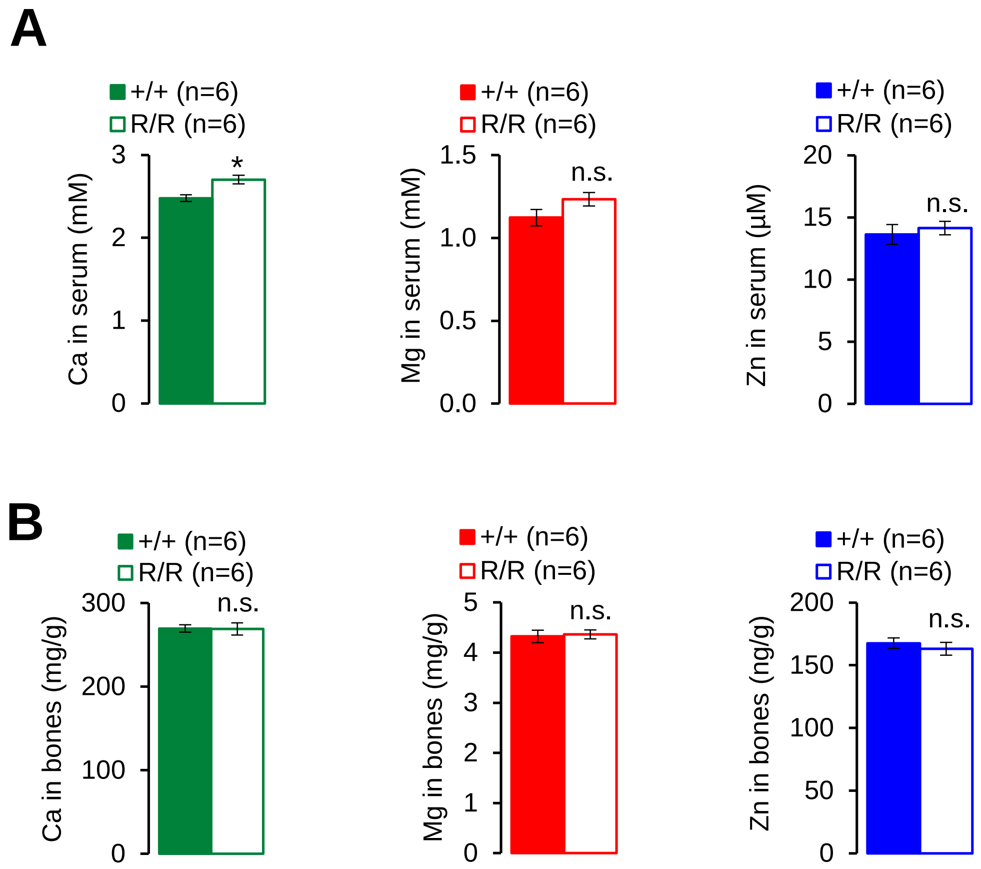

Supplement: Supplementary File [file pnas.1810633116.sapp.pdf]
